# Association Between Risk-of-Bias Assessments and Results of Randomized Trials in Cochrane Reviews: The ROBES Meta-Epidemiologic Study

**DOI:** 10.1093/aje/kwx344

**Published:** 2017-10-19

**Authors:** Jelena Savović, Rebecca M Turner, David Mawdsley, Hayley E Jones, Rebecca Beynon, Julian P T Higgins, Jonathan A C Sterne

**Affiliations:** 1 Population Health Sciences, Bristol Medical School, University of Bristol, Bristol, United Kingdom; 2 National Institute for Health Research; 3 Medical Research Council; 4 MRC Clinical Trials Unit, University College London, London, United Kingdom

**Keywords:** allocation concealment, bias, blinding, meta-analysis, missing data, randomization, randomized trials

## Abstract

Flaws in the design of randomized trials may bias intervention effect estimates and increase between-trial heterogeneity. Empirical evidence suggests that these problems are greatest for subjectively assessed outcomes. For the Risk of Bias in Evidence Synthesis (ROBES) Study, we extracted risk-of-bias judgements (for sequence generation, allocation concealment, blinding, and incomplete data) from a large collection of meta-analyses published in the Cochrane Library (issue 4; April 2011). We categorized outcome measures as mortality, other objective outcome, or subjective outcome, and we estimated associations of bias judgements with intervention effect estimates using Bayesian hierarchical models. Among 2,443 randomized trials in 228 meta-analyses, intervention effect estimates were, on average, exaggerated in trials with high or unclear (versus low) risk-of-bias judgements for sequence generation (ratio of odds ratios (ROR) = 0.91, 95% credible interval (CrI): 0.86, 0.98), allocation concealment (ROR = 0.92, 95% CrI: 0.86, 0.98), and blinding (ROR = 0.87, 95% CrI: 0.80, 0.93). In contrast to previous work, we did not observe consistently different bias for subjective outcomes compared with mortality. However, we found an increase in between-trial heterogeneity associated with lack of blinding in meta-analyses with subjective outcomes. Inconsistency in criteria for risk-of-bias judgements applied by individual reviewers is a likely limitation of routinely collected bias assessments. Inadequate randomization and lack of blinding may lead to exaggeration of intervention effect estimates in randomized trials.

Meta-analyses of randomized trials are often more influential than single trials, and they increasingly inform health-care decisions made by clinicians and health authorities. For their results to be valid, randomized trials should employ rigorous methods that can achieve and preserve comparability of the intervention and control groups (1). For example, concealment of randomized allocation prevents an influence of patient characteristics on allocation to intervention and control groups; blinding of participants and trial personnel prevents differences in patient management between groups; and blinding of outcome assessors prevents knowledge of the assigned intervention group influencing outcome measurement. Randomized trials vary in methodological rigor, and flaws in trial conduct can lead to biased estimation of the intervention effect (2). Systematic reviewers should therefore assess the risk of bias in intervention effect estimates from each included trial.

Meta-epidemiologic studies analyze collections of meta-analyses to provide empirical evidence about the influence of trial design characteristics on trial results (3). Such studies have, however, reached differing conclusions about which trial design characteristics most influence their results (4–8). For example, 4 studies found that lack of adequate allocation concealment was associated with overestimation of treatment effect (9–12), while several other studies did not find evidence for this (4, 5, 13–15). In a previous study, we explored reasons for these discrepancies by combining data from 7 meta-epidemiologic studies (16, 17). To our knowledge, this was the first study to explore the effects of bias on between– and within–meta-analysis heterogeneity using Bayesian hierarchical models. The results suggested that trial results based on subjectively assessed outcomes are more susceptible to bias and that the effect of bias is unpredictable, leading to increased heterogeneity in meta-analyses assessing subjective outcomes (16, 17). Further investigation of the effects of trial characteristics across different interventions, settings, and outcomes in larger collections of meta-analyses (not previously used) may provide more clarity and resolve inconsistencies between previous empirical studies.

Since January 2008, authors of Cochrane reviews have used a “risk-of-bias” tool for assessing included trials (18). The assessors make judgements in relation to “sequence generation,” “allocation concealment,” “blinding of participants, personnel, and outcome assessors,” “incomplete outcome data,” “selective outcome reporting,” and a general category of “other potential threats to validity.” For each of these areas, review authors record whether there was a judgement of low, high, or unclear risk of bias for each trial, together with comments or quotes to justify each judgement. Accumulated standardized risk-of-bias assessments are a potentially useful resource for meta-epidemiologic research.

In this paper, we describe and report the main results from a new, large empirical study investigating the associations of risk-of-bias judgements for sequence generation, allocation concealment, blinding, and incomplete outcome data with treatment effect estimates— the Risk of Bias in Evidence Synthesis (ROBES) Study. Our aims were to examine whether routinely collected risk-of-bias assessments relating to methodological characteristics are associated with effect estimates, to compare these associations with findings from our previous study (17), and to examine further the effect of outcome types in a new collection of meta-analyses.

## METHODS

### Data source

The April 2011 issue of the Cochrane Database of Systematic Reviews (issue 4) included 4,371 intervention reviews (excluding protocols), of which 1,399 had at least 2 completed domains in the Risk of Bias tables. The complete 1,399 reviews were supplied by the Cochrane Informatics and Knowledge Management Department in electronic format, as Review Manager (version 5.0) files (19). We converted these to a customized Microsoft Access database (Microsoft Corporation, Redmond, Washington) using bespoke software which we commissioned from Riskaware Ltd. (Bristol, United Kingdom).

### Data selection and categorization

We selected meta-analyses that fulfilled the following criteria: 1) address a binary outcome; 2) include at least 5 randomized trials, each with at least 1 event across the 2 trial arms; 3) accompanied by risk-of-bias assessments, with all 5 core domains of the tool having been assessed (sequence generation, allocation concealment, blinding, incomplete outcome data, and selective outcome reporting); 4) compare an active intervention with a control or “older” intervention; and 5) include no trials that overlap with another meta-analysis in the data set. Details of the process for selecting eligible meta-analyses are provided in Web Appendix 1 (available at https://academic.oup.com/aje). Meta-analyses can inform estimation of the bias associated with a particular domain only if they contain at least 1 trial at “low risk” of bias and 1 at “high or unclear” risk of bias. We refer to these as *informative* meta-analyses for that bias domain.

We categorized each meta-analysis according to objectivity of the outcome measure (see below), direction of outcome (adverse or favorable) (16, 17), type of intervention (pharmacological, surgical, psychosocial and behavioral, care pathways, or other), clinical area (based on the World Health Organization’s *International Classification of Diseases, Tenth Revision*) (20), and whether the comparator was an active intervention (i.e., not a placebo, untreated, or standard care). Classification of outcome measure objectivity followed the method of Savović et al. (16, 17): We categorized outcome measures as 1) all-cause mortality; 2) other objectively assessed outcome (including live birth, noncephalic birth, low birth weight, miscarriage, pregnancy, and all automated laboratory outcomes); 3) semiobjective outcome (where the outcome event is considered to be measured accurately but the decision behind it is influenced by a clinician’s or patient’s judgement (e.g., hospital admission or readmission, study dropout/withdrawal for any reason, treatment completion, cesarean delivery, spontaneous vaginal birth, operative/assisted delivery, conversion to open surgery, additional treatments administered)); or 4) subjectively assessed outcome (e.g., clinician-assessed outcomes, symptoms and symptom scores, pain, mental health outcomes, cause-specific mortality). Too few meta-analyses had outcomes in the objective and semiobjective categories (categories 2 and 3) for separate analyses to be possible, so we combined these categories as “other objective.” When both objective and subjective methods of outcome assessment were used in different trials contributing to the same meta-analysis, the meta-analysis was categorized as having a subjectively assessed outcome (e.g., some trials in meta-analyses examining smoking cessation used a laboratory measure, while others used patient self-reporting).

### Statistical analysis

To explore correlations between bias domains, we computed odds ratios for the association between risk-of-bias judgements for pairs of domains using logistic regression in Stata 14 (StataCorp LP, College Station, Texas). For the main analyses, we modeled intervention effects as log odds ratios with outcomes coded so that odds ratios less than 1 corresponded to beneficial intervention effects in all meta-analyses. In the main analysis, “high risk” and “unclear risk” bias judgements were grouped together. The underlying idea of the analysis is described in Web Appendix 2 and illustrated in Web Figure 1.

We fitted Bayesian hierarchical bias models, assuming a binomial likelihood (“model 3” by Welton et al. (21)). This assumes random intervention effects (between-trial heterogeneity) within meta-analyses, which allows us to assess whether individual bias domains are associated with increased heterogeneity. The model includes parameters for average bias in intervention effects (log odds ratios comparing trials at “high or unclear” risk of bias with “low” risk of bias, averaged across all meta-analyses) and 2 sources of variation in bias. Variation in bias among trials within meta-analyses was quantified using a κ^2^ term representing the average increase in between-trial heterogeneity in trials at “high or unclear” risk of bias (vs. “low” risk of bias) for each bias domain. Variation in mean bias across meta-analyses was quantified by a measure of between–meta-analysis variance, φ^2^. Posterior mean values for average bias were exponentiated and are reported as the ratio of odds ratios; posterior median values for κ and φ are reported on the log odds ratio scale. All are presented with 95% credible intervals. Meta-analyses containing fewer than 2 studies at “low risk” of bias and at “high or unclear” risk of bias are uninformative for κ and thus were prevented from influencing the estimation of this parameter. Additional statistical analysis information and analysis code is provided in Web Appendix 2.

We conducted univariable analyses for each of 4 risk-of-bias domains (sequence generation, allocation concealment, blinding, and incomplete outcome data) using all informative meta-analyses for that domain (model A in Web Appendix 2). We did not explore the association between the selective outcome reporting domain and intervention effect estimates. This domain currently addresses the nonreporting of outcomes rather than bias in the results available for meta-analysis, so it is not directly relevant to bias in the observed results. Analyses were also stratified according to type of outcome measure (all-cause mortality, other objectively assessed, and subjectively assessed). Multivariable analyses were based on an extended model assuming distinct variance components associated with each bias domain (model B in Web Appendix 2), described elsewhere by Savović et al. (16). We also fitted multivariable analyses that allowed interactions between sequence generation and allocation concealment, allocation concealment and blinding, and sequence generation and blinding (model C in Web Appendix 2). We conducted a univariable sensitivity analysis combining trials with an “unclear” risk-of-bias judgement with those with “low risk” of bias (rather than with “high risk”). We also conducted separate analyses for objective and semiobjective outcomes.

## RESULTS

**Figure 1.**
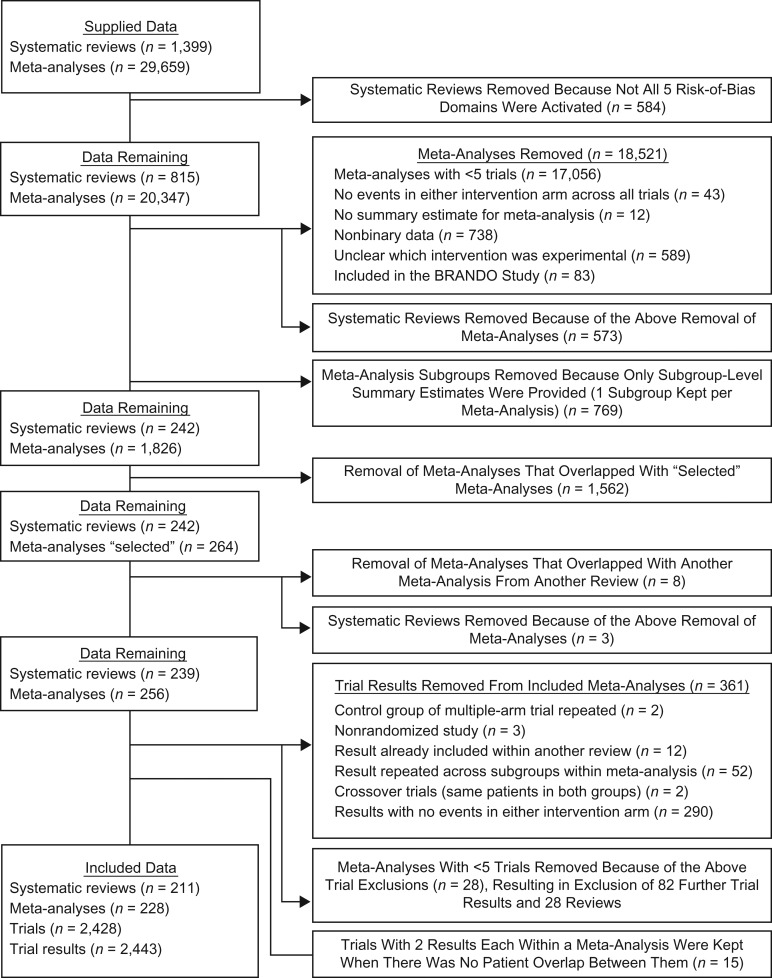
Selection of systematic reviews and meta-analyses from the Cochrane Database of Systematic Reviews (issue 4; April 2011) for inclusion in the ROBES Study. ROBES, Risk of Bias in Evidence Synthesis.

Following our selection process, the final ROBES Study data set consisted of 228 meta-analyses containing 2,443 randomized trials (Figure 1). The full list of included reviews and meta-analysis is provided in Web Appendix 3. The median year of publication of included reviews was 2008 (interquartile range (IQR), 2005–2010; range, 1996–2011), and for trials it was 1999 (IQR, 1992–2005, range, 1950–2011). The median sample size was 1,290 (IQR, 676–3,403; range, 110–341,351) for meta-analyses and 114 (IQR, 60–256; range, 8–182,000) for trials. Based on the categorization of clinical areas in the *International Classification of Diseases, Tenth Revision*, the most frequently assessed conditions were related to pregnancy and childbirth (28 meta-analyses; 12.3%) and mental health (27 meta-analyses; 11.8%), followed by circulatory system conditions (21 meta-analyses; 9.2%) and respiratory system conditions (20 meta-analyses; 8.8%). Subjectively assessed outcomes were reported most frequently, in 127 (55.7%) meta-analyses, followed by all-cause mortality (42 meta-analyses; 18.4%) (Table 1).

**Table 1.** Characteristics of Meta-Analyses and Randomized Trials Included in the ROBES Study, 2011–2015

Characteristic	Meta-Analyses (*n* = 228)		Randomized Trials (*n* = 2,443)	
	No.	%	No.	%
Clinical area, by ICD-10 chapter				
Pregnancy and childbirth	28	12.3	387	15.8
Mental and behavioral disorders	27	11.8	286	11.7
Circulatory system diseases	21	9.2	259	10.6
Respiratory system diseases	20	8.8	196	8.0
Genitourinary system diseases	19	8.3	214	8.8
Perinatal conditions	18	7.9	155	6.3
Digestive system diseases	17	7.5	193	7.9
Infectious and parasitic diseases	11	4.8	113	4.6
Neoplasms	11	4.8	103	4.2
Nervous system diseases	10	4.4	102	4.2
Injury and poisoning	10	4.4	98	4.0
Other ICD-10 chapters	34	14.9	319	13.1
Unclassified	2	0.9	18	0.7
Type of experimental intervention				
Pharmacological	151	66.2	1,688	69.1
Provision of care	14	6.1	111	4.5
Surgical intervention or procedure	12	5.3	126	5.2
Psychosocial and behavioral	11	4.8	125	5.1
Other	40	17.5	393	16.1
Type of comparison intervention				
Pharmacological	26	11.4	251	10.3
Surgical intervention or procedure	8	3.5	99	4.1
Other active intervention	4	1.8	33	1.4
Placebo/no treatment^a^	58	25.4	677	27.7
Placebo	51	22.4	560	22.9
Standard/usual care	32	14.0	307	12.6
No treatment	25	11.0	233	9.5
Standard care/placebo/no treatment^a^	24	10.5	283	11.6
Type of outcome measure^b^				
All-cause mortality	42	18.4	429	17.6
Other objective outcome	20	8.8	197	8.1
Subjective outcome	127	55.7	1,356	55.5
Mixture of objective and subjective outcomes^a^	2	0.9	70	2.9
Semiobjective outcome	37	16.2	391	16.0

Abbreviations: ICD-10, *International Classification of Diseases, Tenth Revision*; ROBES, Risk of Bias in Evidence Synthesis.

^a^ Combined at the meta-analysis level.

^b^ *Other objective outcome*: automated or semiautomated laboratory measures including biochemical measurements and serological tests, birth weight, live birth, preterm birth, clinical pregnancy, unintended pregnancy, and noncephalic birth. *Subjective outcome*: signs and symptoms of disease and improvement thereof, symptom scales and scores, mental health outcomes, imaging and radiological outcomes, pain, quality of life, adverse treatment events, other patient-reported outcomes or those relying on a diagnosis by a physician, and cause-specific deaths. *Mixture of objective and subjective outcomes*: meta-analyses in which some trials used laboratory validation while others used self-reporting for smoking cessation. *Semiobjective outcome* (outcomes for which ascertainment is accurate but their occurrence is influenced by a patient’s or care-provider’s subjective judgement): blood transfusion, prescription of antiplatelet medication, cesarean delivery, spontaneous vaginal birth, preterm birth, oxytocin augmentation, failure of extubation, surgical evacuation, conversion to open surgery, need for further surgery, radical resection, hospital admission, admission to neonatal intensive care unit, hospital readmission, presentation at emergency department, compliance with intervention, completion of the study, withdrawal or dropout from the study, discontinuation of treatment, and not remaining in contact with psychiatric services.

The proportion of trials judged as being at low risk of bias was highest for the incomplete outcome data domain (1,493 trials; 61.1%), followed by sequence generation (1,143 trials; 46.8%), blinding (1,119 trials; 45.8%), and allocation concealment (1,033 trials; 42.3%). The proportion of trials with unclear risk of bias was highest for allocation concealment (1,267 trials; 51.9%) and sequence generation (1,226 trials; 50.2%) and was markedly lower for blinding (641 trials; 26.2%) and incomplete outcome data (580 trials; 23.7%). The proportion of trials rated as being at high risk of bias was highest for blinding (683 trials; 28.0%), followed by incomplete outcome data (370 trials; 15.2%), with low proportions rated as high risk for allocation concealment (143 trials; 5.9%) and sequence generation (74 trials; 3.0%) (Table 2). Numbers of trials with each combination of the 4 risk-of-bias domain judgements are shown by type of outcome in Web Figure 2.

**Table 2.** Numbers and Percentages of Randomized Trials Included in the ROBES Study, by Risk-of-Bias Judgement

Risk-of-Bias Domain	Risk of Bias					
	Low		High		Unclear	
	No.	%	No.	%	No.	%
Sequence generation	1,143	46.8	74	3.0	1,226	50.2
Allocation concealment	1,033	42.3	143	5.9	1,267	51.9
Blinding	1,119	45.8	683	28.0	641	26.2
Incomplete outcome data	1,493	61.1	370	15.2	580	23.7

Abbreviation: ROBES, Risk of Bias in Evidence Synthesis.

For sequence generation, 2,158 trials were included in 189 (82.9%) informative meta-analyses, of which 1,006 (46.6%) were judged as having low risk of bias, 1,081 (50.1%) as having unclear risk of bias, and 71 (3.3%) as having high risk of bias. For allocation concealment, 2,121 trials were included in 188 (82.5%) informative meta-analyses, of which 933 (44.0%) were judged as having low, 1,068 (50.3%) as having unclear, and 120 (5.7%) as having high risk of bias. Only 144 (63.2%) meta-analyses (1,678 trials) were informative for blinding: 854 (50.9%) trials were judged as low, 437 (26.0%) as unclear, and 387 (23.1%) as high risk of bias. For incomplete outcome data, 1,956 trials were included in 167 (73.2%) informative meta-analyses: 1,156 (59.1%) were judged as low, 475 (24.3%) as unclear, and 325 (16.6%) as high risk of bias.

There was a strong association between judgements of low risk of bias for sequence generation and allocation concealment (odds ratio = 10.4, 95% confidence interval: 8.6, 12.5) (Table 3). Odds ratios for this association were consistent across types of outcome variables. Associations between low-risk-of-bias judgements for the other 5 pairs of domains were of smaller magnitude; odds ratios across all trials varied between 1.8 and 2.9 (Table 3).

**Table 3.** Estimated Odds Ratios for the Associations Between Risk-of-Bias Judgements in Randomized Trials Included in the ROBES Study

Risk-of-Bias Domain Pair	All Trials (*n* = 2,443)		All-Cause Mortality (*n* = 429)		Other Objective Outcome (*n* = 197)		“Semiobjective” Outcome^a^ (*n* = 391)		Subjective Outcome^b^ (*n* = 1,426)	
	OR	95% CI	OR	95% CI	OR	95% CI	OR	95% CI	OR	95% CI
Sequence generation, allocation concealment	10.4	8.6, 12.5	11.3	7.1, 17.9	16.7	7.9, 34.9	9.7	6.1, 15.4	9.5	7.4, 12.2
Sequence generation, blinding	2.5	2.2, 3.0	3.1	2.1, 4.6	2.0	1.0, 3.8	2.2	1.5, 3.3	2.8	2.2, 3.4
Sequence generation, incomplete outcome data	2.1	1.8, 2.4	2.7	1.8, 4.0	5.3	2.8, 9.8	1.7	1.1, 2.6	1.8	1.4, 2.2
Allocation concealment, blinding	2.9	2.4, 3.4	4.0	2.7, 6.0	6.0	3.0, 12.1	1.3	0.8, 1.9	3.2	2.6, 4.1
Allocation concealment, incomplete outcome data	2.2	1.8, 2.6	2.9	1.9, 4.4	4.4	2.4, 8.3	1.3	0.9, 2.0	2.0	1.6, 2.5
Blinding, incomplete outcome data	1.8	1.5, 2.1	1.8	1.2, 2.6	1.4	0.7, 2.6	2.1	1.4, 3.2	1.8	1.5, 2.3

Abbreviations: CI, confidence interval; OR, odds ratio; ROBES, Risk of Bias in Evidence Synthesis.

^a^ Outcomes for which ascertainment is accurate but their occurrence is influenced by a patient’s or health-care provider’s subjective judgement (e.g., duration of hospital stay, admissions, withdrawals, cesarean delivery).

^b^ Includes meta-analyses in which some trials had subjective measures and some objective measures (e.g., self-reports and laboratory measures).

Table 4 and Web Figure 3 show results from univariable analyses (based on model A). Intervention effect estimates were exaggerated by an average of 9% in trials judged as being at high or unclear risk of bias for sequence generation (ratio of odds ratios (ROR) = 0.91, 95% credible interval (CrI): 0.86, 0.98). There was only a modest increase in between-trial heterogeneity among such trials compared with trials at low risk of bias (standard deviations (SDs) differed by 0.09 (95% CrI: 0.02, 0.21)). Mean bias varied between meta-analyses, although this variability was imprecisely estimated (SD, 0.10 (95% CrI: 0.02, 0.20); Table 4). There was no convincing evidence that the magnitude of average bias differed according to the type of outcome. Meta-analyses with subjective outcomes contributed the most data to the analysis, and the average bias among these studies was similar to the overall result (ROR = 0.90, 95% CrI: 0.83, 0.98). In multivariable analyses (based on model B), the association between risk-of-bias judgement and intervention effect estimate was attenuated after adjusting for risk-of-bias judgements for allocation concealment, blinding, and incomplete outcome data (ROR = 0.95, 95% CrI: 0.89, 1.03). The average bias was similar across all outcome types (Table 5, Web Figure 4).

**Table 4.** Estimated Ratios of Odds Ratios and Between–Meta-Analysis Heterogeneity in Mean Bias Associated With Risk-of-Bias Judgements in Randomized Trials and Meta-Analyses, by Type of Outcome Measure (Univariable Analyses (Model A)), in the ROBES Study^a^

Risk-of-Bias Domain and Outcome	No. of MAs or RTs Contributing to Analysis		Average Bias		No. of MAs Contributing to κ Estimation	Within-MA Heterogeneity		Between-MA Heterogeneity	
	MAs	RTs	ROR	95% CrI		κ	95% CrI	φ	95% CrI
Sequence generation: high/unclear risk of bias vs. low risk of bias									
All outcomes	189	2,158	0.91	0.86, 0.98	142	0.09	0.02, 0.21	0.10	0.02, 0.20
Mortality	34	363	0.84	0.71, 1.01	27	0.13	0.01, 0.39	0.09	0.01, 0.37
Other objective/semiobjective outcome	47	523	0.99	0.87, 1.16	38	0.10	0.01, 0.31	0.14	0.01, 0.41
Subjective outcome/mixture^b^	108	1,272	0.90	0.83, 0.98	77	0.08	0.01, 0.21	0.08	0.01, 0.22
Allocation concealment: high/unclear risk of bias vs. low risk of bias									
All outcomes	188	2,121	0.92	0.86, 0.98	139	0.05	0.01, 0.15	0.05	0.01, 0.17
Mortality	35	358	0.84	0.71, 1.01	27	0.07	0.01, 0.30	0.12	0.01, 0.42
Other objective/semiobjective outcome	49	524	0.96	0.86, 1.07	40	0.04	0.01, 0.14	0.05	0.01, 0.19
Subjective outcome/mixture	104	1,239	0.91	0.83, 0.99	72	0.08	0.01, 0.25	0.06	0.01, 0.20
Blinding: high/unclear risk of bias vs. low risk of bias									
All outcomes	144	1,678	0.87	0.80, 0.93	105	0.10	0.02, 0.25	0.12	0.02, 0.24
Mortality	31	327	0.83	0.72, 0.97	25	0.06	0.01, 0.26	0.06	0.01, 0.25
Other objective/semiobjective outcome	32	334	0.94	0.81, 1.10	24	0.06	0.01, 0.21	0.06	0.01, 0.28
Subjective outcome/mixture	81	1,017	0.83	0.73, 0.93	56	0.22	0.04, 0.36	0.19	0.03, 0.34
Incomplete outcome data: high/unclear risk of bias vs. low risk of bias									
All outcomes	167	1,956	0.98	0.92, 1.05	112	0.05	0.01, 0.16	0.05	0.01, 0.15
Mortality	29	303	0.92	0.79, 1.08	19	0.08	0.01, 0.32	0.06	0.01, 0.24
Other objective/semiobjective outcome	43	471	1.03	0.90, 1.19	28	0.07	0.01, 0.25	0.06	0.01, 0.25
Subjective outcome/mixture	95	1,182	0.97	0.88, 1.07	65	0.06	0.01, 0.17	0.10	0.01, 0.30

Abbreviations: CrI, credible interval; MA, meta-analysis; ROBES, Risk of Bias in Evidence Synthesis; ROR, ratio of odds ratios; RT, randomized trial.

^a^ For a graphical representation of these results, see Web Figure 3.

^b^ “Mixture” refers to meta-analyses in which some trials had subjective measures and some had objective measures of the same outcome (e.g., self-reports and laboratory measures of smoking cessation).

**Table 5.** Estimated Ratios of Odds Ratios and Between–Meta-Analysis Heterogeneity in Mean Bias Associated With Risk-of-Bias Judgements in Randomized Trials and Meta-Analyses, by Type of Outcome Measure (Multivariable Analyses (Model B)), in the ROBES Study^a^

Risk-of-Bias Domain and Outcome	No. of MAs or RTs Contributing to Analysis		Average Bias		No. of MAs Contributing to κ Estimation	Within-MA Heterogeneity		Between-MA Heterogeneity	
	MAs	RTs	ROR	95% CrI		κ	95% CrI	φ	95% CrI
Sequence generation: high/unclear risk of bias vs. low risk of bias									
All outcomes	189	2,158	0.95	0.88, 1.03	142	0.08	0.02, 0.18	0.11	0.03, 0.22
Mortality	34	363	0.92	0.75, 1.18	27	0.14	0.02, 0.36	0.14	0.03, 0.42
Other objective/semiobjective outcome	47	523	1.06	0.90, 1.28	38	0.14	0.03, 0.33	0.20	0.04, 0.44
Subjective outcome/mixture^b^	108	1,272	0.94	0.84, 1.04	77	0.08	0.02, 0.18	0.11	0.02, 0.24
Allocation concealment: high/unclear risk of bias vs. low risk of bias									
All outcomes	188	2,121	0.96	0.88, 1.03	139	0.06	0.01, 0.15	0.07	0.02, 0.16
Mortality	35	358	0.92	0.74, 1.13	27	0.11	0.03, 0.29	0.15	0.03, 0.42
Other objective/semiobjective outcome	49	524	0.94	0.81, 1.08	40	0.07	0.01, 0.18	0.09	0.02, 0.25
Subjective outcome/mixture	104	1,239	0.95	0.86, 1.07	72	0.10	0.02, 0.23	0.08	0.02, 0.20
Blinding: high/unclear risk of bias vs. low risk of bias									
All outcomes	144	1,678	0.88	0.81, 0.94	105	0.10	0.02, 0.22	0.12	0.03, 0.23
Mortality	31	327	0.87	0.73, 1.03	25	0.10	0.02, 0.26	0.10	0.02, 0.28
Other objective/semiobjective outcome	32	334	0.95	0.79, 1.12	24	0.09	0.02, 0.24	0.10	0.02, 0.34
Subjective outcome/mixture	81	1,017	0.84	0.75, 0.95	56	0.17	0.04, 0.33	0.19	0.05, 0.35
Incomplete outcome data: high/unclear risk of bias vs. low risk of bias									
All outcomes	167	1,956	1.01	0.94, 1.09	112	0.07	0.01, 0.16	0.07	0.02, 0.16
Mortality	29	303	0.99	0.82, 1.18	19	0.11	0.02, 0.31	0.10	0.02, 0.30
Other objective/semiobjective outcome	43	471	1.04	0.90, 1.21	28	0.11	0.02, 0.30	0.09	0.02, 0.26
Subjective outcome/mixture	95	1,182	1.00	0.90, 1.12	65	0.07	0.01, 0.17	0.11	0.03, 0.27

Abbreviations: CrI, credible interval; MA, meta-analysis; ROBES, Risk of Bias in Evidence Synthesis; ROR, ratio of odds ratios; RT, randomized trial.

^a^ For a graphical representation of these results, see Web Figure 4.

^b^ “Mixture” refers to meta-analyses in which some trials had subjective measures and some had objective measures of the same outcome (e.g., self-reports and laboratory measures of smoking cessation).

Because there was a strong association between sequence generation and allocation concealment, the estimates of average bias for these 2 domains may be expected to be similar. Intervention effect estimates were exaggerated by an average of 8% (ROR = 0.92, 95% CrI: 0.86, 0.98) in trials judged to be at high or unclear risk of bias for allocation concealment, but there was very little evidence of an increase in between-trial heterogeneity (SDs differed by 0.05 (95% CrI: 0.01, 0.15)). The variability in average bias across meta-analyses was small (SD, 0.05 (95% CrI: 0.01, 0.17)). There was little evidence that the average bias varied according to type of outcome. Estimates of both between-trial and between–meta-analysis heterogeneity in bias were low for all outcome types. As for sequence generation, the analysis including adjustment for the other 3 domains (model B) produced an attenuated estimate of average bias (ROR = 0.96, 95% CrI: 0.88, 1.03), and the estimates were very similar across all outcome types (Table 5, Web Figure 4).

Intervention effect estimates were exaggerated by an average of 13% (ROR = 0.87, 95% CrI: 0.80, 0.93) in trials judged to be at high or unclear risk of bias for blinding. Between-trial heterogeneity was modestly increased for such studies (SDs differed by 0.10 (95% CrI: 0.02, 0.25)), and average bias varied between meta-analyses (SD, 0.12 (95% CrI: 0.02, 0.24)). There was little evidence that intervention effects differed according to type of outcome. Increases in between-trial heterogeneity (SDs differed by 0.22 (95% CrI: 0.04, 0.36)) and between–meta-analysis heterogeneity in average bias (SD, 0.19 (95% CrI: 0.03, 0.34)) appeared greater in meta-analyses assessing subjective outcomes than for all-cause mortality or other objective outcomes. In adjusted analysis (model B), the estimated effect of high or unclear risk of bias due to blinding was similar to the unadjusted estimate (ROR = 0.88, 95% CrI: 0.81, 0.94).

There was little evidence that intervention effects were exaggerated in trials judged to be at high or unclear risk of bias for incomplete outcome data (ROR = 0.98, 95% CrI: 0.92, 1.05). The corresponding estimated increase in between-trial heterogeneity was small (SDs differed by 0.05 (95% CrI: 0.01, 0.15)). There was little evidence that average bias or increases in between-trial heterogeneity varied according to type of outcome. The adjusted estimates were very similar to the unadjusted estimates (Table 5, Web Figure 4).

The results of the sensitivity analysis (model A) in which trials with an unclear risk-of-bias judgement were combined with those at low risk of bias are shown in Web Table 1. The average intervention effects in meta-analyses with high risk of bias for blinding compared with those with low or unclear risk of bias were exaggerated, on average, by 13% (ROR = 0.87, 95% CrI: 0.79, 0.95), consistent with the main analysis. For the other 3 bias domains, the 95% credible intervals for estimates of average bias included the null. These analyses included fewer informative meta-analyses, especially for sequence generation and allocation concealment, and consequently the estimates had wider credible intervals. Estimated increases in between-trial heterogeneity were larger for sequence generation, compared with those observed in the main analysis.

The separate estimates for subgroups of meta-analyses with “other objective” and “semiobjective” outcomes (which were analyzed together in the main analysis) were similar to each other for allocation concealment and blinding. They differed somewhat for sequence generation (ROR = 0.85 (95% CrI: 0.67, 1.09) for other objective outcomes and ROR = 1.08 (95% CrI: 0.91, 1.34) for semiobjective outcomes) and incomplete outcome data (ROR = 0.94 (95% CrI: 0.72, 1.22) for other objective outcomes and ROR = 1.11 (95% CrI: 0.93, 1.30) for semiobjective outcomes), but the credible intervals were wide and overlapping (Web Table 2).

In multivariable models with interaction terms (model C), an interaction was observed between allocation concealment and blinding (ROR = 0.84, 95% CrI: 0.74, 0.96) and between sequence generation and blinding (ROR = 0.77, 95% CrI: 0.66, 0.91) (Web Table 3). This means that lack of blinding may introduce greater bias in estimation of intervention effects within studies with inadequate randomization than within studies with adequate randomization.

## DISCUSSION

Using a collection of 2,443 randomized trials included in 228 meta-analyses, our estimates of the association between average intervention effect estimates and routinely collected risk-of-bias judgements for sequence generation, allocation concealment, blinding, and incomplete outcome data confirm that problems with randomization and a lack of blinding are, on average, associated with a modest (around 10%) exaggeration of treatment effect estimates. Lack of blinding appears to have the largest influence on treatment effect estimates, and this remains after adjustment for other domains. There was little evidence that these biases varied according to the type of outcome measure assessed. Although there were some differences in the ratios of odds ratios for different outcome types in univariable analyses, the 95% credible intervals overlapped, and the differences were attenuated or disappeared in adjusted analyses. We found little evidence that trials assessed as being at high or unclear risk of bias for incomplete outcome data produced systematically different estimates compared with trials at low risk of bias for this domain, for all types of outcome measures. Variability of treatment effects was higher in trials that lacked blinding and had subjective outcomes, suggesting that for such trials the direction and magnitude of bias is unpredictable. Such variability in bias was observed both between trials within a meta-analysis and across meta-analyses. There was little evidence of such variation in bias for other bias domains or for objectively determined outcomes. Multivariable analyses suggested that effects of individual risk-of-bias domain judgements were less than additive, in that estimated effects of 2 bias domain judgements together were less than the combined individual effects.

To our knowledge, this study represents the most comprehensive attempt to date to quantify the influence of 4 bias domains on intervention effect estimates from randomized controlled trials using routinely collected risk-of-bias assessments from published Cochrane reviews. Our findings indicate that assessments are associated with effect sizes, on average, for 3 of the 4 domains, providing some degree of validation of the risk-of-bias tool. However, to interpret our findings as evidence of bias due to the methods implemented in the trials, it is important to consider the accuracy and reliability of these risk-of-bias assessments. The assessments were made by a large number of Cochrane review authors with varying degrees of experience and training, and we did not replicate assessments to determine how appropriate they were. Although detailed guidance on how to assess risk of bias in trials included in Cochrane reviews is available in chapter 8 of the Cochrane Handbook (18), review authors have reported that they find aspects of the assessment difficult (22). Indeed, some studies have found that the assessor agreement and interrater reliability of the risk-of-bias tool is suboptimal (23, 24). Specifically, individual reviewers have different criteria for judging a study to be at “low risk” of bias: Some may be more confident about making a judgement with less information, while others would opt for “unclear risk.” Standard advice is that 2 assessors independently assess risk of bias and resolve disagreements through discussion. We presume that this advice was followed. As a safeguard that recommended assessment methods were followed, at least to some extent, we restricted eligibility to reviews that had completed all 5 prescribed bias domains. It is possible that individual review teams had their own criteria for rating a study “low-risk” for each of the domains, which may have differed from those described in the handbook.

In our main analyses, risk-of-bias judgements were dichotomized so that “high” risk and “unclear” risk were considered together. This allows for like-for-like comparisons with results from most of the previous empirical studies, including our previous study (17). Furthermore, there were few “high-risk-of-bias” judgements, so analyses with the alternative dichotomization of “high” versus “low” or “unclear” risk of bias were not informative. For the domains of sequence generation and allocation concealment, a “high-risk-of-bias” judgement was recorded in only 3% and 6% of trials, respectively (Table 2). We demonstrated that Cochrane assessors frequently reach a judgement of “unclear” risk of bias (Table 2). Inadequate reporting of key features of trial design is a likely explanation for this high rate of uncertainty, particularly for methods of sequence generation and allocation concealment. This observation is consistent with findings from a study by Turner et al. (25) that allocation concealment was reported in sufficient detail in 30% (722/2,396) of published randomized trials.

Our adjusted results for sequence generation and allocation concealment were largely consistent with meta-analyses of all previous meta-epidemiologic studies reported in a recent systematic review (26). Blinding and incomplete data in studies included in that review were not assessed in the same way as in our study and cannot be meaningfully compared with our results. Our results for average bias were slightly smaller than those from our previous study, the Bias in Randomized and Observational Studies (BRANDO) Study (17). This may reflect dilution due to measurement error, arising because the risk-of-bias assessments in the current study were conducted by a heterogeneous group of Cochrane reviewers. In contrast, assessments used in the BRANDO Study were done by teams of trained methodologists, and data were only combined in the BRANDO analyses where the definitions for adequate versus inadequate study method were consistent across studies. Our finding that the lack of blinding in trials with subjective outcomes can lead to biased effect estimates, but the direction and magnitude of such bias are unpredictable, also confirms a finding from the BRANDO Study (16, 17). The main difference between findings from the current study and those from the BRANDO Study is that here we do not see a clear difference in the magnitude of bias according to type of outcome.

In summary, our results confirm that some aspects of the conduct of randomized trials, particularly blinding, are associated with a modest exaggeration of treatment effects on average, but there is little evidence that the average bias differs according to whether the outcome was subjectively or objectively assessed. However, lack of blinding in trials with subjective outcomes leads to increased heterogeneity and hence unpredictable bias in effect estimates. As far as possible, clinical and policy decisions should be cautious when they are based on trials in which blinding was not reported or not feasible and outcome measures were subjectively assessed. Future development of tools for assessing risk of bias in randomized trials (27, 28) should reflect this observation and collect information on the subjectivity of an outcome. Facilities for capture of detailed routine assessments of risk of bias in randomized trials should be made available for future meta-epidemiologic research and could contribute to further improvement in methods of risk-of-bias assessment.

## Supplementary Material

Web Material

## Abbreviations

BRANDO: Bias in Randomized and Observational Studies
CrI: credible interval
IQR: interquartile range
MRC: Medical Research Council
ROBES: Risk of Bias in Evidence Synthesis
ROR: ratio of odds ratios
SD: standard deviation
