# Supplementary material for: Association Between Risk-of-Bias Assessments and Results of Randomized Trials in Cochrane Reviews: The ROBES Meta-Epidemiologic Study
Source: Am J Epidemiol. 2017 Oct 19;187(5):1113–22. doi: 10.1093/aje/kwx344 (PMC5928453; doi:10.1093/aje/kwx344)
Supplement: Web Material [file kwx344savovicwebmaterialfinal.pdf]

“Association Between Risk-of-Bias Assessments and Results of Randomized Trials in Cochrane Reviews: the ROBES Meta-Epidemiologic Study”

Web Material

Table of Contents

List of Web Figures and Tables 2

Abbreviations 2

Web Appendix 1: Supplementary Methods 3

Selection of eligible meta-analyses 3

Web Appendix 2: Analysis Details, Model Specification and WinBUGS Code 5

Illustration of the analysis on a single meta-analysis example 5

Statistical analysis details 6

WinBUGS code for univariable analyses (Model A) for estimation of average bias and between and within meta-analysis heterogeneity 10

WinBUGS code for multivariable analyses (Model B) for estimation of average bias and between and within meta-analysis heterogeneity 12

WinBUGS code for multivariable analyses with interaction terms (Model C), allowing for interactions between sequence generation, allocation concealment and blinding 15

Web Appendix 3: Included Reviews and Meta-Analyses 20

Web Figure 2 35

Web Figure 3 36

Web Figure 4 37

Web Table 1 38

Web Table 2 39

Web Table 3 40

List of Web Figures and Tables

Web Figure 1. Example Meta-analysis, Depicting the Ratio of Odds Ratios Comparing the Overall Intervention Effect in Studies at High or Unclear Risk of Bias With That in Studies at Low Risk of Bias for Sequence Generation..6

Web Figure 2. Number of Trials With Each Combination of the Four Risk of Bias Domain Judgements by Type of Outcome Measure 35

Web Figure 3. Estimated Ratios of Odds Ratios and Effects on Heterogeneity Associated With Risk of Bias Judgements for Each Domain Independently, According to Type of Outcome Measure: Univariable Analyses (Model A)...36

Web Figure 4. Estimated Ratios of Odds Ratios and Effects on Heterogeneity From Multivariable Analyses of Associations With Risk of Bias Judgements for Each Domain, Adjusted for the Effect of the Other Three Domains (Model B) 37

Web Table 1. Estimated Ratios of Odds Ratios and Between-Meta-Analysis Heterogeneity in Mean Bias Associated With Risk of Bias Judgements, According to Type of Outcome Measure: Univariable Sensitivity Analyses for High Risk of Bias Compared to Low or Unclear Risk of Bias .38

Web Table 2. Estimated Ratios of Odds Ratios and Between-Meta-Analysis Heterogeneity in Mean Bias Associated With Risk of Bias Judgements, According to Type of Outcome Measure: Univariable Sensitivity Analyses for Meta-Analyses With Other Objective and Semi-Objective Outcomes and for High Risk of Bias Compared to Low Or Unclear Risk of Bias 39

Web Table 3. Estimated Ratios of Odds Ratios and Between-Meta-Analysis Heterogeneity in Mean Bias Associated With Risk of Bias Judgments, According to Type of Outcome Measure: Multivariable Analyses With Interactions...40

Abbreviations

(The) BRANDO study – study name (BRANDO - Bias in Randomized and Observational studies)

(The) ROBES study – study name (ROBES - Risk of Bias in Evidence Synthesis)

Web Appendix 1: Supplementary Methods

Selection of eligible meta-analyses

We carried out study selection using a combination of semi-automated queries and manual data categorization by the study team.

Mapping of the risk of bias items: Within the Review Manager software, authors of Cochrane reviews can amend the wording of standard risk of bias items or can add and exclude specific items. All verbatim 'non-standard' risk of bias domains were classified into categories to avoid loss of usable risk of bias data due to differences in wording. For example, if the user-defined bias domain was labelled 'randomization method' this was classified as 'sequence generation' domain. The vast majority of risk of bias tables did however contain the standard wording for domains.

Exclusion of ineligible data: From the initial dataset of 1399 reviews we excluded reviews that did not have all five recommended risk of bias items completed: sequence generation, allocation concealment, blinding (assessed just as 'blinding' without further descriptors or as blinding of specific, reviewer-defined, groups such as patients or assessors), incomplete outcome data and selective reporting. Having a completed 'Other bias' domain was not required as the use of this domain is recommended for potential topic- or design-specific biases that are not necessarily relevant to all types of studies. We further excluded meta-analyses that had fewer than 5 included trials and meta-analyses without a summary estimate, labelled 'non-estimable' in the supplied dataset (e.g. due to zero events in both intervention groups across all studies, or where review authors chose not to calculate a summary estimate (e.g. where meta-analysis was not considered appropriate)). We further excluded meta-analyses with continuous outcomes. The remaining meta-analyses were then scrutinized individually as follows.

Selection of meta-analysis with primary outcomes: In each of the remaining reviews, we tagged meta-analyses with outcome(s) that were described as primary outcomes in the text of the review. The first meta-analysis from each review to be included in our study (referred to as the 'selected' meta-analysis) was selected as follows: 1) If, having followed the exclusion process described above, only one meta-analysis in the review had a primary outcome then this was selected; 2) if there were multiple primary outcome meta-analyses available, the meta-analysis with the most included trials (and, if tied, that with most included participants) was selected from among these; and 3) if there were no primary outcome meta-analyses in the review, the meta-analysis with the most included trials (and, if tied, that with most included participants) was selected.

Exclusion of overlapping meta-analyses: For each review, we then examined whether there were any overlapping trials between the 'selected' meta-analysis and all other meta-analyses in that

review, in the order of decreasing size (number of trials, then number of participants), starting with meta-analyses with primary outcomes. The additional meta-analysis was included in the study only if it did not contain any trials already included in the selected meta-analysis. Any subsequent meta-analysis from the same review could only be included if it had no overlapping trials with any of the already included meta-analyses and so on.

Dealing with meta-analyses' subgroups: Meta-analyses that had no subgroups were taken whole and all related trial outcome level data were included. Of the meta-analyses that had subgroups, some had an overall estimate across all subgroups, while others only provided estimates for individual subgroups. In the case of the former, we treated such meta-analyses in the same way as those that did not have subgroups, ignoring the subgroups. In a small number of meta-analyses, results from one trial were recorded across 2 subgroups. We checked these to ensure that the same participants did not contribute to both estimates. If overlap was identified (e.g. 3-arm trial where the comparison group was used in both subgroup analyses), we removed one of the occurrences of a trial, at random. When only subgroup level estimates were provided, we assumed there was a justifiable rationale for not calculating an overall estimate (e.g. important clinical heterogeneity). In such cases we included only the largest subgroup, by number of included trials, if tied by number of participants, and if still tied, the subgroup that appeared first in the review.

Meta-analyses categorized as having an active comparison where it was not clear which intervention was experimental or novel were excluded. For the remaining meta-analyses comparing two active interventions, the newer or the more recently introduced intervention was coded as experimental intervention and the older or standard intervention was coded as a comparator in the analysis.

Web Appendix 2: Analysis Details, Model Specification and WinBUGS Code

Illustration of the analysis on a single meta-analysis example

The underlying idea of the analysis is illustrated in Web Figure 1, for a single meta-analysis of antihypertensive medication for prevention of cardiovascular mortality and morbidity in the elderly.(3) In this example, the overall odds ratio in studies assessed as at high or unclear risk of bias for sequence generation was 0.69 (95% confidence interval 0.61, 0.78), while the corresponding odds ratio for studies at low risk of bias was 0.87 (95% confidence interval 0.65, 1.16). The ratio of odds ratios comparing studies at high/unclear with low risk of bias measures the difference in effect size in the two sets of trials, and was 0.79 (=0.69/0.87) with 95% confidence interval 0.60 to 1.02 (estimated using meta-regression in Stata 14). This process is then repeated for each included meta-analysis and average ratio of odds ratio is estimated across all meta-analyses and measures of how bias varies across meta-analyses.

Web Figure 1. Example Meta-analysis, Depicting the Ratio of Odds Ratios Comparing the Overall Intervention Effect in Studies at High or Unclear Risk of Bias With That in Studies at Low Risk of Bias for Sequence Generation

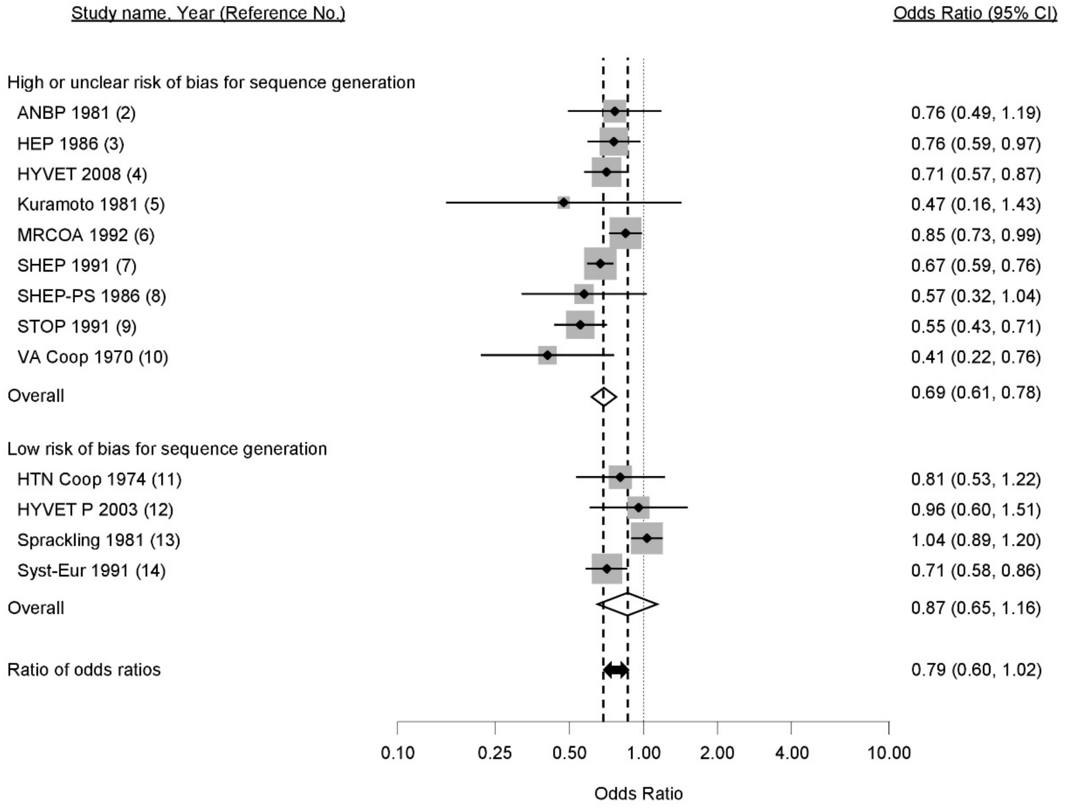

Random-effects meta-analysis of antihypertensive medication (vs placebo or no treatment) for cardiovascular mortality and morbidity, stratified by risk of bias for sequence generation. Odds ratios smaller than 1 favor antihypertensive medication and larger than 1 favor placebo or no treatment. The solid line represents "no difference" between treatments, and the dashed lines represent the estimates from the subgroup meta-analyses of studies with high or unclear risk of bias (top panel) and studies with low risk of bias (lower panel) for sequence generation. The double arrow shows the difference between the subgroup estimates, which is quantified using meta-regression to calculate a ratio of odds ratios and its confidence interval. CI, confidence interval.

Statistical analysis details

Datasets for main analyses were prepared, and the analyses of correlations between risk of bias domains were carried out using Stata 14 statistical software. Bayesian hierarchical models were fitted in WinBUGS, using two chains run for 500,000 iterations after a burn-in of 50,000 iterations, with the exception of multivariable analyses with interactions, for which two chains were run for 100,000 iterations following a burn-in of 100,000 iterations.

In all models, we assumed that the observed number of events r_{im0}, r_{im1} in each treatment arm of trial *i* in meta-analysis *m* has a binomial distribution:

$$r_{im0} \sim Binomial(\pi_{im0}, n_{im0})$$

$$r_{im1} \sim Binomial(\pi_{im1}, n_{im1})$$

$$logit(\pi_{im0}) = \mu_{im}$$

$$logit(\pi_{im1}) = \mu_{im} + \theta_{im}$$

In the univariable analyses, the underlying log-odds ratio θ_{im} in trial *i* in meta-analysis *m* was assumed equal to

$$\theta_{im} = \delta_{im} + \beta_{im} C_{im} \qquad \text{[Model A, univariable analyses]}$$

where $C_{im} = 1$ for trials with high or unclear risk of bias and $C_{im} = 0$ for trials with low risk of bias for each bias domain. The parameter δ_{im} represents the intervention effect in trials with low risk of bias. These are assumed to be randomly distributed within each meta-analysis *m:*

$$\delta_{im} \sim Normal(d_m, \tau_m^2) \qquad \textit{(i)}$$

Parameter β_{im} quantifies the potential bias associated with the study design characteristic of interest in trial *i* within meta-analysis *m*. We assumed the following model structure, which allows the bias to vary within each meta-analysis and also allows average bias b_m to vary across meta-analyses:

$$\beta_{im} \sim Normal(b_m, \kappa^2) \qquad \textit{(ii)}$$

$$b_m \sim Normal(b_0, \varphi^2)$$

For all location parameters (overall mean bias b_0, trial baseline response rates μ_{im}, average intervention effects d_m), vague Normal(0,1000) or Normal(0,100) priors were assumed. A generic informative prior was declared for the between-trial heterogeneity variances (based on external empirical data) (15): $log(\tau_m^2) \sim Normal(-2.56, 1.74^2)$. Modified inverse gamma(0.001,0.001) priors were declared for κ and φ, as used in the BRANDO study (16).

For multivariable analyses without interactions, we specified Model B for the underlying log odds ratio θ_{im}:

$$\theta_{im} = \delta_{im} + \beta_{1im} C_{1im} + \beta_{2im} C_{2im} + \beta_{3im} C_{3im} + \beta_{4im} C_{4im} \qquad \text{[Model B, multivariable analysis]}$$

where C_{1im} to C_{4im} refer to bias domains: sequence generation, allocation concealment, blinding and incomplete outcome data; and β_{1im} *to* β_{4im} quantify the corresponding potential biases associated with high or unclear risk of bias judgements for these domains. The δ_{im} were assumed randomly distributed within each meta-analysis, as in (i), and a separate model of the form (ii) was assumed for each bias parameter β_{1im} *to* β_{4im}.

For multivariable analyses with interaction terms, allowing for interactions between sequence generation, allocation concealment and blinding, we specified Model C for the underlying log odds ratio θ_{im}:

$$\theta_{im} = \delta_{im} + \beta_{1im}C_{1im} + \beta_{2im}C_{2im} + \beta_{3im}C_{3im} + \beta_{4im}C_{4im} + \gamma_{1im}C_{2im}C_{3im} + \gamma_{2im}C_{1im}C_{2im} + \gamma_{3im}C_{1im}C_{3im}$$

[Model C, multivariable analysis with interactions]

where parameters γ_{1im} *to* γ_{3im} quantify the interactions between: allocation concealment and blinding, sequence generation and allocation concealment, and sequence generation and blinding, respectively, when both domains within their respective interaction pairs are at high or unclear risk of bias. The δ_{im} were assumed randomly distributed within each meta-analysis, as in (i), and a separate model of the form (ii) was assumed for each bias parameter β_{1im} to β_{4im} and for each interaction parameter γ_{1im} to γ_{3im}.

Priors for all location parameters and for between-trial heterogeneity variances τ_m^2 were identical to those used in the univariable models. In the multivariable models, we used narrower modified inverse gamma(0.01,0.01) priors for the κ and φ parameters. This change was made because of convergence difficulties with the inverse gamma(0.001,0.001) priors.

References

1. Musini VM, Tejani AM, Bassett K, et al. Pharmacotherapy for hypertension in the elderly. *Cochrane Database Syst Rev* 2009(4):CD000028.
2. Trial Management Committee. Treatment of mild hypertension in the elderly. A study initiated and administered by the National Heart Foundation of Australia. *Med J Aust* 1981;2(8):398-402.
3. Coope J, Warrender TS. Randomized trial of treatment of hypertension in elderly patients in primary care. *BMJ* 1986;293(6555):1145-1151.
4. Beckett NS, Peters R, Fletcher AE, et al. Treatment of hypertension in patients 80 years of age or older. *N Engl J Med* 2008;358(18):1887-1898.
5. Kuramoto K, Matsushita S, Kuwajima I, et al. Prospective study on the treatment of mild hypertension in the aged. *Jpn Heart J* 1981;22(1):75-85.
6. Meade TW, Peart S, Brennan PJ, et al. Medical-Research-Council trial of treatment of hypertension in older adults - principal results. *BMJ* 1992;304(6824):405-412.

7. Probstfield JL. Prevention of stroke by antihypertensive drug-treatment in older persons with isolated systolic hypertension - final results of the Systolic Hypertension in the Elderly Program (SHEP). *JAMA* 1991;265(24):3255-3264.
8. Perry HM, McDonald RH, Hulley SB, et al. Systolic Hypertension in the Elderly Program, Pilot-Study (SHEP-PS) - morbidity and mortality experience. *J Hypertens* 1986;4:S21-S23.
9. Dahlof B, Lindholm LH, Hansson L, et al. Morbidity and mortality in the Swedish trial in old patients with hypertension (STOP-Hypertension). *Lancet* 1991;338(8778):1281-1285.
10. Veterans Administration Cooperative Study Group on Antihypertensive Agents. Effects of treatment on morbidity in hypertension. II. Results in patients with diastolic blood pressure averaging 90 through 114 mm Hg. *JAMA* 1970;213(7):1143-1152.
11. Hypertension-Stroke Cooperative Study Group. Effect of antihypertensive treatment on stroke recurrence. *JAMA* 1974;229(4):409-418.
12. Bulpitt CJ, Beckett NS, Cooke J, et al. Results of the pilot study for the Hypertension in the Very Elderly Trial. *J Hypertens* 2003;21(12):2409-2417.
13. Sprackling ME, Mitchell JRA, Short AH, et al. Blood-pressure reduction in the elderly - a randomized controlled trial of methyldopa. *BMJ* 1981;283(6300):1151-1153.
14. Amery A, Birkenhager W, Bulpitt CJ, et al. Syst-Eur. A multicentre trial on the treatment of isolated systolic hypertension in the elderly: objectives, protocol, and organization. *Aging (Milano)* 1991;3(3):287-302.
15. Turner RM, Davey J, Clarke MJ, et al. Predicting the extent of het erogeneity in meta-analysis, using empirical data from the Cochrane Database of Systematic Reviews. *Int J Epidemiol* 2012;41(3):818-827.
16. Savović J, Jones H, Altman D, et al. Influence of reported study design characteristics on intervention effect estimates from randomised controlled trials: combined analysis of meta-epidemiological studies. *Health Technol Assess* 2012;16(35):1-82.

WinBUGS code for univariable analyses (Model A) for estimation of average bias and between and within meta-analysis heterogeneity

```
model{
        for (i in 1:N) {
        r[i] ~ dbin(p[i],n[i])                                    # likelihood
        logit(p[i]) <- mu[s[i]] +  treat[i]*(delta[i] + beta[i] *C1[i])      # model
          beta[i]~dnorm(b[ma[i]],p.k2[ma[i]])I(-10,10)            # between study, within meta-analysis, variation in bias
          delta[i]~dnorm(d[ma[i]],p.d[ma[i]])I(-10,10)            #RE for treatment effect within meta-analysis
     rhat[i] <- p[i] * n[i]                                                  #calculate residual deviance
     dev[i] <- 2 * (r[i] * (log(r[i])-log(rhat[i]))  +  (n[i]-r[i]) * (log(n[i]-r[i]) - log(n[i]-rhat[i])))
     redundant[i] <- C0[i]
                }
                resdev <- sum(dev[])

        for (j in 1:N_trial) {mu[j] ~ dnorm(0,.01)}               # priors for study baseline effects - unrelated

        for (m in 1:N_ma) {
                d[m] ~ dnorm(0,.01)                   # priors for true fixed (unrelated) treatment effects
          b[m] ~ dnorm(b0,p.phi)                              #between meta-analysis variation in mean bias
                var_d[m]~dlnorm(-2.56,0.33)           # generic informative prior for between-trial variances
                p.d[m] <- 1/var_d[m]
                p.k2[m] <- p.k*equals(kappa_ok[m],1) + cut(p.k)*equals(kappa_ok[m],0)
        }

        b0 ~ dnorm(0,.001)                                        # vague prior for overall mean bias

   p.k1 ~ dgamma(.001,.001)                                       # vague prior for between study variation in bias
        kappa <- pow(p.k,-0.5)
   p.k <- p.k1/(1-patom.k)
   patom.k ~ dbeta(1,1)

        p.phi1 ~ dgamma(.001,.001)                    #vague prior for between meta-analysis variation in mean bias
        phi <- pow(p.phi,-0.5)
        p.phi <- p.phi1/(1-patom.phi)
        patom.phi ~ dbeta(1,1)
```

```
    # Parameters to monitor
    q[1] <- b0
    q[2] <- exp(b0)
    q[3] <- kappa
    q[4] <- phi

}
```

WinBUGS code for multivariable analyses (Model B) for estimation of average bias and between and within meta-analysis heterogeneity

```
model{
        for (i in 1:N) {
        r[i] ~ dbin(p[i],n[i])                                  # likelihood

        logit(p[i]) <- mu[s[i]] +  treat[i]*(delta[i]  + beta1[i]*seq1[i] + beta2[i]*alloc1[i] + beta3[i]*blind1[i] + beta4[i]*incomp1[i])     # model

          beta1[i]~dnorm(b1[ma[i]],p.ka1[ma[i]])I(-10,10)      # between study, within MA, variation in bias
          beta2[i]~dnorm(b2[ma[i]],p.ka2[ma[i]])I(-10,10)      # between study, within MA, variation in bias
          beta3[i]~dnorm(b3[ma[i]],p.ka3[ma[i]])I(-10,10)      # between study, within MA, variation in bias
          beta4[i]~dnorm(b4[ma[i]],p.ka4[ma[i]])I(-10,10)      # between study, within MA, variation in bias

          delta[i]~dnorm(d[ma[i]],p.d[ma[i]])I(-10,10)          # RE for treatment effect within meta-analysis
     rhat[i] <- p[i] * n[i]                                          # calculate residual deviance
     dev[i] <- 2 * (r[i] * (log(r[i])-log(rhat[i]))  +  (n[i]-r[i]) * (log(n[i]-r[i]) - log(n[i]-rhat[i])))
                }
                resdev <- sum(dev[])

        for (j in 1:N_trial) {mu[j] ~ dnorm(0,.01)}                 # priors for study baseline effects - unrelated


        for (m in 1:N_ma) {
                d[m] ~ dnorm(0,.01)                     # priors for true fixed (unrelated) treatment effects
                b1[m] ~ dnorm(b01,p.phi1)               # between meta-analysis variation in mean bias
                b2[m] ~ dnorm(b02,p.phi2)               # between meta-analysis variation in mean bias
                b3[m] ~ dnorm(b03,p.phi3)               # between meta-analysis variation in mean bias
        b4[m] ~ dnorm(b04,p.phi4)                       # between meta-analysis variation in mean bias
     var_d[m]~dlnorm(-2.56,0.33)                                # generic informative prior for between-trial variances
                p.d[m]<- 1/var_d[m]
                p.ka1[m] <- p.k1*equals(kappa_ok1[m],1) + cut(p.k1)*equals(kappa_ok1[m],0)
        p.ka2[m] <- p.k2*equals(kappa_ok2[m],1) + cut(p.k2)*equals(kappa_ok2[m],0)
                p.ka3[m] <- p.k3*equals(kappa_ok3[m],1) + cut(p.k3)*equals(kappa_ok3[m],0)
                p.ka4[m] <- p.k4*equals(kappa_ok4[m],1) + cut(p.k4)*equals(kappa_ok4[m],0)
        }
```

```
b01 ~ dnorm(0,.001)                        # vague prior for overall mean bias
b02 ~ dnorm(0,.001)                        # vague prior for overall mean bias
b03 ~ dnorm(0,.001)                        # vague prior for overall mean bias
b04 ~ dnorm(0,.001)                        # vague prior for overall mean bias


p.kz1~dgamma(.01,.01)
kappa1 <- pow(p.k1,-0.5)
p.k1<-p.kz1/(1-patom.k1)
patom.k1~dbeta(1,1)

p.kz2~dgamma(.01,.01)
kappa2 <- pow(p.k2,-0.5)
p.k2<-p.kz2/(1-patom.k2)
patom.k2~dbeta(1,1)

p.kz3~dgamma(.01,.01)
kappa3 <- pow(p.k3,-0.5)
p.k3<-p.kz3/(1-patom.k3)
patom.k3~dbeta(1,1)

p.kz4~dgamma(.01,.01)
kappa4 <- pow(p.k4,-0.5)
p.k4<-p.kz4/(1-patom.k4)
patom.k4~dbeta(1,1)


p.phiz1~dgamma(.01,.01)
phi1 <- pow(p.phi1,-0.5)
p.phi1<-p.phiz1/(1-patom.phi1)
patom.phi1~dbeta(1,1)

p.phiz2~dgamma(.01,.01)
phi2 <- pow(p.phi2,-0.5)
p.phi2<-p.phiz2/(1-patom.phi2)
patom.phi2~dbeta(1,1)
```

```
        p.phiz3~dgamma(.01,.01)
        phi3 <- pow(p.phi3,-0.5)
        p.phi3<-p.phiz3/(1-patom.phi3)
        patom.phi3~dbeta(1,1)

        p.phiz4~dgamma(.01,.01)
        phi4 <- pow(p.phi4,-0.5)
        p.phi4<-p.phiz4/(1-patom.phi4)
        patom.phi4~dbeta(1,1)

        # Parameters to monitor
        qf[1] <- b01
        qf[2] <- b02
        qf[3] <- b03
        qf[4] <- b04
        qf[5] <- exp(b01)
        qf[6] <- exp(b02)
        qf[7] <- exp(b03)
        qf[8] <- exp(b04)

        qr[1] <- kappa1
        qr[2] <- kappa2
        qr[3] <- kappa3
        qr[4] <- kappa4
        qr[5] <- phi1
        qr[6] <- phi2
        qr[7] <- phi3
        qr[8] <- phi4
}
```

WinBUGS code for multivariable analyses with interaction terms (Model C), allowing for interactions between sequence generation, allocation concealment and blinding

```
model{
    for (i in 1:N) {
    r[i] ~ dbin(p[i],n[i])                                    # likelihood

#model
    logit(p[i]) <- mu[s[i]] +  treat[i]*(delta[i]  + beta1[i]*seq1[i] + beta2[i]*alloc1[i] + beta3[i]*blind1[i] + beta4[i]*incomp1[i]       +
gamma[i,1]*alloc1[i]*blind1[i] + gamma[i,2]*alloc1[i]*seq1[i] + gamma[i,3]*blind1[i]*seq1[i])

      beta1[i]~dnorm(b1[ma[i]],p.ka1[ma[i]])I(-10,10)     # between study, within MA, variation in bias
      beta2[i]~dnorm(b2[ma[i]],p.ka2[ma[i]])I(-10,10)     # between study, within MA, variation in bias
      beta3[i]~dnorm(b3[ma[i]],p.ka3[ma[i]])I(-10,10)     # between study, within MA, variation in bias
      beta4[i]~dnorm(b4[ma[i]],p.ka4[ma[i]])I(-10,10)     # between study, within MA, variation in bias

    for(z in 1:3){
                gamma[i,z]~dnorm(bi[ma[i],z], pi.ka[ma[i],z])I(-10,10)
            }

      delta[i]~dnorm(d[ma[i]],p.d[ma[i]])I(-10,10)          # RE for treatment effect within meta-analysis
     rhat[i] <- p[i] * n[i]                                       # calculate residual deviance
     dev[i] <- 2 * (r[i] * (log(r[i])-log(rhat[i]))  +  (n[i]-r[i]) * (log(n[i]-r[i]) - log(n[i]-rhat[i])))
            }
            resdev <- sum(dev[])

    for (j in 1:N_trial) {mu[j] ~ dnorm(0,.01)}                   # priors for study baseline effects - unrelated


    for (m in 1:N_ma) {
            d[m] ~ dnorm(0,.01)                           # priors for true fixed (unrelated) treatment effects
            b1[m] ~ dnorm(b01,p.phi1)                     # between meta-analysis variation in mean bias
            b2[m] ~ dnorm(b02,p.phi2)                     # between meta-analysis variation in mean bias
            b3[m] ~ dnorm(b03,p.phi3)                     # between meta-analysis variation in mean bias
    b4[m] ~ dnorm(b04,p.phi4)                             # between meta-analysis variation in mean bias
```

```
		for(z in 1:3){
			bi[m, z] ~ dnorm(bi0[z], pi.phi[z])
		}

	  var_d[m]~dlnorm(-2.56,0.33)				# generic informative prior for between-trial variances
		p.d[m]<- 1/var_d[m]
		p.ka1[m] <- p.k1*equals(kappa_ok1[m],1) + cut(p.k1)*equals(kappa_ok1[m],0)
	p.ka2[m] <- p.k2*equals(kappa_ok2[m],1) + cut(p.k2)*equals(kappa_ok2[m],0)
		p.ka3[m] <- p.k3*equals(kappa_ok3[m],1) + cut(p.k3)*equals(kappa_ok3[m],0)
		p.ka4[m] <- p.k4*equals(kappa_ok4[m],1) + cut(p.k4)*equals(kappa_ok4[m],0)

	# Only informative if both interacting variables informative
		pi.ka[m,1]<-pi.k[1]*equals(kappa_ok2[m],1)*equals(kappa_ok3[m],1) + cut(pi.k[1])*(1-
(equals(kappa_ok2[m],1)*equals(kappa_ok3[m],1)))
		pi.ka[m,2]<-pi.k[2]*equals(kappa_ok2[m],1)*equals(kappa_ok1[m],1) + cut(pi.k[2])*(1-
(equals(kappa_ok2[m],1)*equals(kappa_ok1[m],1)))
		pi.ka[m,3]<-pi.k[3]*equals(kappa_ok3[m],1)*equals(kappa_ok1[m],1) + cut(pi.k[3])*(1-
(equals(kappa_ok3[m],1)*equals(kappa_ok1[m],1)))

		}


	b01 ~ dnorm(0,.001)			# vague prior for overall mean bias
	b02 ~ dnorm(0,.001)			# vague prior for overall mean bias
	b03 ~ dnorm(0,.001)			# vague prior for overall mean bias
	b04 ~ dnorm(0,.001)			# vague prior for overall mean bias

	for(z in 1:3){
		bi0[z] ~ dnorm(0,.001)
	}

	p.kz1~dgamma(.01,.01)
	kappa1 <- pow(p.k1,-0.5)
	p.k1<-p.kz1/(1-patom.k1)
	patom.k1~dbeta(1,1)

	p.kz2~dgamma(.01,.01)
```

```
kappa2 <- pow(p.k2,-0.5)
p.k2<-p.kz2/(1-patom.k2)
patom.k2~dbeta(1,1)

p.kz3~dgamma(.01,.01)
kappa3 <- pow(p.k3,-0.5)
p.k3<-p.kz3/(1-patom.k3)
patom.k3~dbeta(1,1)

p.kz4~dgamma(.01,.01)
kappa4 <- pow(p.k4,-0.5)
p.k4<-p.kz4/(1-patom.k4)
patom.k4~dbeta(1,1)

for(z in 1:3){
        pi.kz[z]~dgamma(.01,.01)
        kappai[z]<-pow(pi.k[z],-0.5)
        pi.k[z]<-pi.kz[z]/(1-i.patom.k[z])
        i.patom.k[z]~dbeta(1,1)
}


p.phiz1~dgamma(.01,.01)
phi1 <- pow(p.phi1,-0.5)
p.phi1<-p.phiz1/(1-patom.phi1)
patom.phi1~dbeta(1,1)

p.phiz2~dgamma(.01,.01)
phi2 <- pow(p.phi2,-0.5)
p.phi2<-p.phiz2/(1-patom.phi2)
patom.phi2~dbeta(1,1)

p.phiz3~dgamma(.01,.01)
phi3 <- pow(p.phi3,-0.5)
p.phi3<-p.phiz3/(1-patom.phi3)
patom.phi3~dbeta(1,1)
```

```
p.phiz4~dgamma(.01,.01)
phi4 <- pow(p.phi4,-0.5)
p.phi4<-p.phiz4/(1-patom.phi4)
patom.phi4~dbeta(1,1)

for(z in 1:3){
	pi.phiz[z]~dgamma(.01,.01)
	i.phi[z]<-pow(pi.phi[z],-0.5)
	pi.phi[z]<-pi.phiz[z]/(1-i.patom.phi[z])
	i.patom.phi[z]~dbeta(1,1)
}

# Parameters to monitor
qf[1] <- b01
qf[2] <- b02
qf[3] <- b03
qf[4] <- b04
qf[5] <- exp(b01)
qf[6] <- exp(b02)
qf[7] <- exp(b03)
qf[8] <- exp(b04)

for(z in 1:3){
	exp.bi0[z]<-exp(bi0[z])
}

bias.alloc.blind<-b02+b03+bi0[1]
bias.alloc.seq<-b02+b03+bi0[2]
bias.blind.seq<-b03+b01+bi0[3]

ror.bias.alloc.blind<-exp(bias.alloc.blind)
ror.bias.alloc.seq<-exp(bias.alloc.seq)
ror.bias.blind.seq<-exp(bias.blind.seq)

qr[1] <- kappa1
qr[2] <- kappa2
qr[3] <- kappa3
```

```
    qr[4] <- kappa4
    qr[5] <- phi1
    qr[6] <- phi2
    qr[7] <- phi3
    qr[8] <- phi4

}
```

Web Appendix 3: Included Reviews and Meta-Analyses

Cochrane review number	Review Title	Cochrane Library Issue; Year	Review DOI	MA No.*	Experimental intervention	Comparison intervention	Outcome
CD000012	Alternative versus conventional institutional settings for birth	Issue 1; 2005	10.1002/14651858.CD000012.pub2	1.06	Alternative birth setting	Conventional birth setting	Spontaneous vaginal birth
CD000019	Thyrotropin-releasing hormone added to corticosteroids for women at risk of preterm birth for preventing neonatal respiratory disease	Issue 2; 2004	10.1002/14651858.CD000019.pub2	1.01	TRH+steroids	Steroids	Death prior to hospital discharge
CD000023	Antibiotics for sore throat	Issue 4; 2006	10.1002/14651858.CD000023.pub3	1.04	Antibiotics	Placebo	Incidence of otitis media within 14 days. Otitis media defined by clinical diagnosis
CD000028	Pharmacotherapy for hypertension in the elderly	Issue 4; 2009	10.1002/14651858.CD000028.pub2	1.02	Antihypertensive drug therapy	Placebo/no treatment	Cardiovascular mortality and morbidity
CD000032	Energy and protein intake in pregnancy	Issue 4; 2003	10.1002/14651858.CD000032	2.02	Balanced protein/energy supplementation	No supplementation	Preterm birth
CD000051	Cephalic version by postural management for breech presentation	Issue 3; 2000	10.1002/14651858.CD000051	1.01	Cephalic version by postural management	No treatment	Non-cephalic births
CD000059	Clozapine versus typical neuroleptic medication for schizophrenia	Issue 1; 2009	10.1002/14651858.CD000059.pub2	1.03	Clozapine	Typical drugs	Global impression: 1. Not clinically improved
CD000083	External cephalic version for breech presentation at term	Issue 1; 1996	10.1002/14651858.CD000083	1.01	External cephalic version	No treatment	Non-cephalic births
CD000088	Family intervention for schizophrenia	Issue 4; 2006	10.1002/14651858.CD000088.pub2	1.04	Family intervention	Standard care	Global state: 1. Relapse
CD000110	Hospitalisation and bed rest for multiple pregnancy	Issue 3; 1997	10.1002/14651858.CD000110	1.09	Hospitalisation for bed rest	No hospitalisation	Low birthweight (< 2500 g)
CD000139	Prophylactic methylxanthines for endotracheal extubation in preterm infants	Issue 12; 2010	10.1002/14651858.CD000139.pub2	1.01	Methylxanthine	Placebo/no treatment	Failed extubation
CD000140	Methylxanthine treatment for apnoea in preterm infants	Issue 12; 2010	10.1002/14651858.CD000140.pub2	1.01	Methylxanthine	Placebo/no treatment	Failed apnoea reduction after 2 - 7 days
CD000164	Phenobarbital prior to preterm birth for preventing neonatal periventricular haemorrhage	Issue 1; 2010	10.1002/14651858.CD000164.pub2	1.03	Phenobarbital	Control	Perinatal mortality
CD000174	Prophylactic intravenous indomethacin for preventing mortality and morbidity in preterm infants	Issue 3; 2002	10.1002/14651858.CD000174	1.12	Prophylactic indomethacin	Placebo or no treatment	Severe IVH (grades III - IV)

CD000198	Support during pregnancy for women at increased risk of low birthweight babies	Issue 3; 2003	10.1002/14651858.CD000198	1.06	Additional support	Usual care	Gestational age < 37 weeks at birth
CD000209	Vitamin E for neuroleptic-induced tardive dyskinesia	Issue 4; 2001	10.1002/14651858.CD000209	1.06	Vitamin E	Placebo	Any adverse effect
CD000215	Anthelmintics for people with neurocysticercosis	Issue 1; 2010	10.1002/14651858.CD000215.pub3	1.12	Albendazole	No anthelmintic	Seizures during treatment
CD000229	Vitamin K prior to preterm birth for preventing neonatal periventricular haemorrhage	Issue 1; 2010	10.1002/14651858.CD000229.pub2	1.03	Vitamin K	Control	Severe (grades 3 and 4) PVH
CD000243	Antibiotics for acute maxillary sinusitis	Issue 2; 2008	10.1002/14651858.CD000243.pub2	1.01	Antibiotics	Placebo	Clinical failure defined as a lack of cure or improvement at 7 to 15 days of follow up
CD000245	Antibiotics for acute bronchitis	Issue 4; 2004	10.1002/14651858.CD000245.pub2	3.01	Antibiotic	Placebo	Number of patients with productive cough
CD000247	Antibiotics for the common cold and acute purulent rhinitis	Issue 3; 2005	10.1002/14651858.CD000247.pub2	1.01	Antibiotic	Placebo	Persisting symptoms 1 to 7 days
CD000313	Discharge planning from hospital to home	Issue 1; 2004	10.1002/14651858.CD000313.pub2	2.04	Discharge planning	Standard care with no structured discharge planning	Unscheduled readmission within 3 months of discharge from hospital
CD000329	Endometrial resection and ablation versus hysterectomy for heavy menstrual bleeding	Issue 2; 1999	10.1002/14651858.CD000329	1.05	TCRE/ablation	Hysterectomy	Proportion requiring further surgery for HMB
CD000361	Intravenous immunoglobulin for preventing infection in preterm and/or low birth weight infants	Issue 1; 2004	10.1002/14651858.CD000361.pub2	1.01	IVIG	Placebo/no treatment	Sepsis, one or more episodes
CD000425	Speech and language therapy for aphasia following stroke	Issue 5; 2010	10.1002/14651858.CD000425.pub2	1.13	Speach & Language Therapy (SLT)	No SLT	Number of drop-outs (any reason)
CD000479	Surgery or embolization for varicoceles in subfertile men	Issue 1; 2009	10.1002/14651858.CD000479.pub4	1.01	Varicocele oclusion	No treatment	Pregnancy rate
CD000509	Inhaled nitric oxide for respiratory failure in preterm infants	Issue 3; 2007	10.1002/14651858.CD000509.pub3	1.01	Inhaled NO	Placebo/no treatment	Death before discharge
CD000511	Prophylactic animal derived surfactant extract for preventing morbidity and mortality in preterm infants	Issue 4; 1997	10.1002/14651858.CD000511	1.08	Animal derived surfactant extract	Placebo	Effect on bronchopulmonary dysplasia
CD000941	Vaginal misoprostol for cervical ripening and induction of labour	Issue 1; 2003	10.1002/14651858.CD000941	1.06	misoprostol	placebo	Oxytocin augmentation
CD000978	Interventions for preventing oral mucositis for patients with cancer receiving treatment	Issue 12; 2010	10.1002/14651858.CD000978.pub4	3.03	Amifostine	Placebo/no treatment	Mucositis (severe)
CD000978	Interventions for preventing oral mucositis for patients with cancer receiving treatment	Issue 12; 2010	10.1002/14651858.CD000978.pub4	8.03	GM-CSF	Placebo/no treatment	Mucositis (severe)
CD000978	Interventions for preventing oral mucositis for patients with cancer receiving treatment	Issue 12; 2010	10.1002/14651858.CD000978.pub4	12.02	Keratinocyte GF	Placebo	Mucositis (moderate plus severe)

CD000994	Home care by outreach nursing for chronic obstructive pulmonary disease	Issue 3; 2001	10.1002/14651858.CD000994	1.02	Respiratory outreach nurse	Routine care	Mortality
CD001008	Hypnotherapy for smoking cessation	Issue 2; 1998	10.1002/14651858.CD001008	2.01	Hypnotherapy	Attention/Advice	smoking cessation at 6m+ follow up
CD001055	Interventions for promoting smoking cessation during pregnancy	Issue 3; 1998	10.1002/14651858.CD001055.pub2	1.01	All interventions to promote cessation	Control (routine care/placebo)	Continued smoking in late pregnancy
CD001058	Antibiotics for preterm rupture of membranes	Issue 2; 2003	10.1002/14651858.CD001058	4.01	Antibiotics	No antibiotic	Perinatal death/death before discharge
CD001059	Calcium supplementation during pregnancy for preventing hypertensive disorders and related problems	Issue 8; 2010	10.1002/14651858.CD001059.pub3	1.02	Calcium	Placebo	Pre-eclampsia
CD001079	Prophylactic protein free synthetic surfactant for preventing morbidity and mortality in preterm infants	Issue 1; 2010	10.1002/14651858.CD001079.pub2	1.01	Prophylactic synthetic surfactant	Placebo	Neonatal mortality
CD001100	Fixed dose subcutaneous low molecular weight heparins versus adjusted dose unfractionated heparin for venous thromboembolism	Issue 9; 2010	10.1002/14651858.CD001100.pub3	1.02	LMWH	UFH	Incidence of recurrent venous thromboembolism at the end of follow up
CD001180	Therapeutic ultrasound for venous leg ulcers	Issue 1; 2008	10.1002/14651858.CD001180.pub2	2.01	High frequency ultrasound	no ultrasound	Proportion of ulcers completely healed during study follow up (varying durations of follow up)
CD001218	Acupuncture for migraine prophylaxis	Issue 1; 2009	10.1002/14651858.CD001218.pub2	2.01	Acupuncture	Sham accupuncture	Response
CD001239	Intravenous immunoglobulin for suspected or subsequently proven infection in neonates	Issue 1; 2004	10.1002/14651858.CD001239.pub2	1.01	IVIG	Placebo/no treatment	Mortality from any cause
CD001266	Bronchodilators for bronchiolitis	Issue 3; 2006	10.1002/14651858.CD001266.pub2	1.04	Bronchodilator	Placebo	Hospital admission after treatment (outpatients)
CD001302	Intra-venous fluids for the prevention of severe ovarian hyperstimulation syndrome	Issue 2; 2002	10.1002/14651858.CD001302	1.01	IV fluid	Placebo/no treatment	Severe OHSS incidence per woman randomized
CD001364	Zinc for the common cold	Issue 2; 2011	10.1002/14651858.CD001364.pub3	2.13	Zinc supplement	Placebo	Bad taste
CD001390	Injectable vaccines for preventing pneumococcal infection in patients with chronic obstructive pulmonary disease	Issue 4; 2006	10.1002/14651858.CD001390.pub2	1.01	Pneumococcal Vaccine	Placebo/no treatment	Pneumonia
CD001396	Selective serotonin reuptake inhibitors for premenstrual syndrome	Issue 4; 2002	10.1002/14651858.CD001396	1.11	SSRIs	Placebo	Study withdrawal
CD001423	Serenoa repens for benign prostatic hyperplasia	Issue 2; 2009	10.1002/14651858.CD001423.pub2	1.06	SR	Placebo	)

CD001446	Corticosteroids for Guillain-Barre syndrome	Issue 2; 2010	10.1002/14651858.CD001446.pub3	1.02	CS	Control	Improvement by one or more grades after four weeks
CD001501	Endometrial resection / ablation techniques for heavy menstrual bleeding	Issue 4; 2009	10.1002/14651858.CD001501.pub3	13.02	Second generation ENDOMETRIAL ABLATION	First generation ENDOMETRIAL ABLATION	Satisfaction rate
CD001502	Intra-uterine insemination versus fallopian tube sperm perfusion for non-tubal infertility	Issue 3; 2004	10.1002/14651858.CD001502.pub2	1.07	Fallopian tube sperm perfusion	Intrauterine insemination	Sensitivity analysis: pregnancy rate per couple for non tubal subfertility (any duration of infertility)
CD001546	Laparoscopic versus open surgery for suspected appendicitis	Issue 4; 2004	10.1002/14651858.CD001546.pub2	1.01	Laparoscopic appendectomy	Conventional appendectomy	Wound infections
CD001735	Support surfaces for pressure ulcer prevention	Issue 4; 2008	10.1002/14651858.CD001735.pub3	6.01	Alternating-pressure	constant low-pressure	Pressure ulcer incidence
CD001867	Opioid antagonists for alcohol dependence	Issue 1; 2005	10.1002/14651858.CD001867.pub2	1.01	Naltrexone	Placebo	Return to heavy drinking
CD001869	Antiviral treatment for Bell's palsy (idiopathic facial paralysis)	Issue 4; 2009	10.1002/14651858.CD001869.pub4	1.01	Antivirals plus corticosteroid or placebo	corticosteroid or placebo	Incomplete recovery at end of study
CD001894	Assisted hatching on assisted conception (IVF and ICSI)	Issue 2; 2009	10.1002/14651858.CD001894.pub4	1.01	Assisted hatching	No treatment	Live birth per woman randomised
CD001942	Corticosteroids for Bell's palsy (idiopathic facial paralysis)	Issue 2; 2009	10.1002/14651858.CD001942.pub3	1.01	Corticosteroids	Placebo/no treatment	Incomplete recovery six months or more after randomisation
CD001955	Glucocorticoids for croup	Issue 1; 2000	10.1002/14651858.CD001955.pub2	1.11	Glucocorticoid	Placebo	Return visits and/or (re)admissions by glucocorticoid
CD002024	Alpha 2 -adrenergic agonists for the management of opioid withdrawal	Issue 2; 2009	10.1002/14651858.CD002024.pub3	2.06	Adrenergic	Methadone	Completion of treatment
CD002025	Buprenorphine for the management of opioid withdrawal	Issue 2; 2006	10.1002/14651858.CD002025.pub3	2.06	Buprenorphine	Clonidine	Number completing withdrawal treatment
CD002041	Hyperbaric oxygen for carbon monoxide poisoning	Issue 1; 2005	10.1002/14651858.CD002041.pub2	1.01	Hyperbaric Oxygen (HBO)	Normobaric Oxygen (NBO)	Presence of symptoms or signs at time of primary analysis (4-6 weeks)
CD002047	Leflunomide for the treatment of rheumatoid arthritis	Issue 1; 2003	10.1002/14651858.CD002047	16.09	leflunomide	MTX	alopecia, leflunomide vs. MTX
CD002063	Intravenous immunoglobulin for Guillain-Barre syndrome	Issue 1; 2006	10.1002/14651858.CD002063.pub3	2.02	IVIg	Plasma Exchange	Number improved by 1 or more disability grades after 4 weeks
CD002120	Oral contraceptive pill for primary dysmenorrhoea	Issue 2; 2009	10.1002/14651858.CD002120.pub2	1.01	Combined OCP	placebo or no treatment	Pain improvement

CD002130	Platelet glycoprotein IIb/IIIa blockers during percutaneous coronary intervention and as the initial medical treatment of non-ST segment elevation acute coronary syndromes	Issue 4; 2001	10.1002/14651858.CD002130	1.01	Platelet glycoprotein IIb/IIIa blockers	Placebo or usual care	30-day mortality
CD002130	Platelet glycoprotein IIb/IIIa blockers during percutaneous coronary intervention and as the initial medical treatment of non-ST segment elevation acute coronary syndromes	Issue 4; 2001	10.1002/14651858.CD002130	8.01	Platelet glycoprotein Iib/IIIa blockers	Placebo	30-day mortality
CD002243	Corticosteroids for treating severe sepsis and septic shock	Issue 1; 2004	10.1002/14651858.CD002243.pub2	1.01	Steroids	Standard therapy or placebo	28-day all-cause mortality
CD002250	Prophylactic antibiotic administration during second and third trimester in pregnancy for preventing infectious morbidity and mortality	Issue 4; 2002	10.1002/14651858.CD002250	1.04	Prophylactic antibiotics	Placebo	Preterm delivery
CD002294	Interventions for preoperative smoking cessation	Issue 7; 2010	10.1002/14651858.CD002294.pub3	1.01	Any intervention to promote cessation	Standard care	Smoking cessation at time of surgery
CD002770	Mechanical ventilation for newborn infants with respiratory failure due to pulmonary disease	Issue 4; 2002	10.1002/14651858.CD002770	1.01	Mechanical ventilation	No mechanical ventilation (rescue MV allowed)	Any reported mortality
CD002771	Kangaroo mother care to reduce morbidity and mortality in low birthweight infants	Issue 2; 2003	10.1002/14651858.CD002771	1.04	Kangaroo mother care	conventional neonatal care	Mortality at latest follow up
CD002773	Neuromuscular paralysis for newborn infants receiving mechanical ventilation	Issue 2; 2005	10.1002/14651858.CD002773.pub2	1.03	Routine paralysis	no/selective paralysis	Pneumothorax (with or without pulmonary interstitial emphysema)
CD002787	Inhaled nitric oxide for acute respiratory distress syndrome (ARDS) and acute lung injury in children and adults	Issue 7; 2010	10.1002/14651858.CD002787.pub2	1.01	Inhaled nitric oxide	Placebo or no intervention	Longest follow up mortality (complete case analysis): INO vs. control
CD002837	Enteral versus parenteral nutrition for acute pancreatitis	Issue 1; 2010	10.1002/14651858.CD002837.pub2	1.01	Enteral Nutrition	Total Parenteral nutrition	Mortality
CD002865	Mifepristone for induction of labour	Issue 4; 2000	10.1002/14651858.CD002865	1.03	Mifepristone	placebo/no treatment	Caesarean section
CD002897	Intraoperative Mitomycin C for glaucoma surgery	Issue 4; 2005	10.1002/14651858.CD002897.pub2	1.01	Intraoperative Mitomycin C	Placebo or no treatment	Failure at 12 months
CD002898	Antiviral treatment and other therapeutic interventions for herpes simplex virus epithelial keratitis	Issue 1; 2008	10.1002/14651858.CD002898.pub3	1.1	Acyclovir	Idoxuridine	Acyclovir versus idoxuridine: 7-day & 14-day healing
CD002898	Antiviral treatment and other therapeutic interventions for herpes simplex virus epithelial keratitis	Issue 1; 2008	10.1002/14651858.CD002898.pub3	1.15	Acyclovir	Vidarabine	Acyclovir versus vidarabine: 7-day & 14-day healing
CD002898	Antiviral treatment and other therapeutic interventions for herpes simplex virus epithelial keratitis	Issue 1; 2008	10.1002/14651858.CD002898.pub3	1.22	Ganciclovir	Acyclovir	Ganciclovir versus acyclovir: 7-day & 14-day healing
CD002907	Antibiotic prophylaxis for cirrhotic patients with upper gastrointestinal bleeding	Issue 2; 2002	10.1002/14651858.CD002907	1.03	Antibiotic	Control	Bacterial infections

CD002958	Early postnatal discharge from hospital for healthy mothers and term infants	Issue 3; 2002	10.1002/14651858.CD002958	1.06	Early discharge	Standard discharge	Proportion of women not breastfeeding in first eight weeks postpartum
CD003000	Education interventions for adults who attend the emergency room for acute asthma	Issue 3; 2007	10.1002/14651858.CD003000.pub2	1.04	Education	usual care	Presentation at emergency department (end of follow up)
CD003053	Insulin-sensitising drugs (metformin, rosiglitazone, pioglitazone, D-chiro-inositol) for women with polycystic ovary syndrome, oligo amenorrhoea and subfertility	Issue 1; 2010	10.1002/14651858.CD003053.pub4	1.03	Metformin	placebo or no treatment	Ovulation rate
CD003101	Vaginal prostaglandin (PGE2 and PGF2a) for induction of labour at term	Issue 4; 2003	10.1002/14651858.CD003101	1.02	Prostaglandin E2	Placebo/No treatment	Uterine hyperstimulation with FHR changes
CD003162	The Epley (canalith repositioning) manoeuvre for benign paroxysmal positional vertigo	Issue 2; 2004	10.1002/14651858.CD003162.pub2	1.01	Epley Manoeuvre	Placebo Manoeuvre	Subjective report of complete symptom resolution
CD003246	Intravenous oxytocin alone for cervical ripening and induction of labour	Issue 3; 2001	10.1002/14651858.CD003246	1.03	IV oxytocin	Placebo or exp management	Caesarean section
CD003281	Stimulation of the wrist acupuncture point P6 for preventing postoperative nausea and vomiting	Issue 2; 2009	10.1002/14651858.CD003281.pub3	1.02	Acupoint P6 stimulation	Sham	Vomiting
CD003331	Exercise based rehabilitation for heart failure	Issue 3; 2004	10.1002/14651858.CD003331.pub2	1.01	All exercise interventions	Usual care	All cause mortality up to12 month follow up
CD003368	Addition of drug/s to a chemotherapy regimen for metastatic breast cancer	Issue 11; 2010	10.1002/14651858.CD003368.pub3	4.01	Control	Addition of a drug	Treatment-related deaths (all trials)
CD003481	Ibuprofen for the treatment of patent ductus arteriosus in preterm and/or low birth weight infants	Issue 1; 2008	10.1002/14651858.CD003481.pub3	2.01	Ibuprofen	Indomethacin	Failure to close a PDA (after single or three doses)
CD003499	Antiepileptics for aggression and associated impulsivity	Issue 4; 2008	10.1002/14651858.CD003499.pub2	6.01	Any epileptic drug	Placebo	Non-compliance: leaving the study early, any reason
CD003598	Antihypertensive treatment for kidney transplant recipients	Issue 3; 2009	10.1002/14651858.CD003598.pub2	1.03	CCB	Placebo/no treatment	Graft loss at last follow-up
CD003626	Bile acids for primary sclerosing cholangitis	Issue 2; 2003	10.1002/14651858.CD003626	1.01	UDCA	placebo or no treatment	Mortality at the end of treatment
CD003666	Volume-targeted versus pressure-limited ventilation in the neonate	Issue 3; 2005	10.1002/14651858.CD003666.pub2	1.01	Volume targeted (newer technology)	Pressure limited	Death in hospital
CD003670	Gowning by attendants and visitors in newborn nurseries for prevention of neonatal morbidity and mortality	Issue 3; 2003	10.1002/14651858.CD003670	1.04	No gown	Gown	Nasal colonisation
CD003677	Surgical approach to hysterectomy for benign gynaecological disease	Issue 3; 2009	10.1002/14651858.CD003677.pub4	2.05	Laparoscopic Hysterectomy	Abdominal Hysterectomy	Intraoperative visceral injury (dich)

CD003678	Pre and post-operative medical therapy for endometriosis surgery	Issue 3; 2004	10.1002/14651858.CD003678.pub2	2.05	Post-surgical medical therapy	placebo or no treatment	Pregnancy
CD003719	Recombinant versus urinary human chorionic gonadotrophin for final oocyte maturation triggering in IVF and ICSI cycles	Issue 2; 2005	10.1002/14651858.CD003719.pub2	1.01	recombinant hCG	urinary hCG	Ongoing pregnancy / live birth rate per woman
CD003766	Continuous support for women during childbirth	Issue 3; 2007	10.1002/14651858.CD003766.pub2	1.05	Continuous support	Usual care	Spontaneous vaginal birth
CD003840	Eradication therapy for peptic ulcer disease in Helicobacter pylori positive patients	Issue 2; 2006	10.1002/14651858.CD003840.pub4	1.01	duodenal ulcer acute healing with H. pylori eradication + ulcer healing drug	ulcer healing drug alone	Proportion not healed
CD003897	Interleukin 2 receptor antagonists for kidney transplant recipients	Issue 1; 2010	10.1002/14651858.CD003897.pub3	1.04	IL2Ra	Placebo/no treatment	Acute rejection: clinically suspected or biopsy proven
CD004004	Radiotherapy for neovascular age-related macular degeneration	Issue 4; 2004	10.1002/14651858.CD004004.pub2	1.02	Radiation therapy	No radiation	Three or more lines visual acuity lost at 12 months
CD004015	Lay health workers in primary and community health care for maternal and child health and the management of infectious diseases	Issue 1; 2005	10.1002/14651858.CD004015.pub2	2.01	Lay Health Workers	Usual care	Initiated Breastfeeding
CD004046	Acupuncture for depression	Issue 2; 2005	10.1002/14651858.CD004046.pub2	2.02	Electro-acupunture	Antidepressants (Sbgrp1: Amitriptyline)	Improvement in depression
CD004064	Chemotherapy for advanced gastric cancer	Issue 2; 2005	10.1002/14651858.CD004064.pub2	2.02	Combination Chemo	Single-agent therapy	Tumour response
CD004064	Chemotherapy for advanced gastric cancer	Issue 2; 2005	10.1002/14651858.CD004064.pub2	6.02	Docetaxel regimens	Non-Docetaxel regimens	Tumour response
CD004073	Vitamin supplementation for preventing miscarriage	Issue 2; 2005	10.1002/14651858.CD004073.pub2	1.02	Vitamin(s)	No vitamins	Early or late miscarriage
CD004075	Fetal pulse oximetry for fetal assessment in labour	Issue 2; 2007	10.1002/14651858.CD004075.pub3	3.25	Fetal pulse oximetry (FPO) + conventional fetal monitoring (CTG)	CTG only	Admission to neonatal intensive care unit
CD004115	Rectal 5-aminosalicylic acid for induction of remission in ulcerative colitis	Issue 2; 2003	10.1002/14651858.CD004115	1.01	Rectal 5-ASA	Placebo	Symptomatic Improvement
CD004147	Psychosocial combined with agonist maintenance treatments versus agonist maintenance treatments alone for treatment of opioid dependence	Issue 4; 2008	10.1002/14651858.CD004147.pub3	1.01	Any Psychosocial+pharm	Pharm standard	Retention in treatment
CD004275	Additional bedtime H2-receptor antagonist for the control of nocturnal gastric acid breakthrough	Issue 4; 2009	10.1002/14651858.CD004275.pub3	1.01	H2-receptor antagonist	No treatment	Prevalence rate of nocturnal gastric acid breakthrough
CD004332	Acamprosate for alcohol dependence	Issue 9; 2010	10.1002/14651858.CD004332.pub2	1.01	Acamprosate	Placebo	Return to any drinking
CD004343	Ethamsylate for the prevention of morbidity and mortality in preterm or very low birth weight infants	Issue 3; 2003	10.1002/14651858.CD004343	1.02	Ethamsylate	Placebo	Mortality to hospital discharge

CD004388	Human recombinant activated protein C for severe sepsis	Issue 1; 2008	10.1002/14651858.CD004388.pub3	1.09	APC	Placebo	Serious bleeding events (days 0 to 28)
CD004401	Antibiotics for the prevention of acute and chronic suppurative otitis media in children	Issue 4; 2006	10.1002/14651858.CD004401.pub2	1.01	Antibiotic	Placebo/no treatment/innefective treatment	Prevention - any AOM or CSOM during intervention
CD004405	Corticosteroids for acute bacterial meningitis	Issue 9; 2010	10.1002/14651858.CD004405.pub3	1.01	Corticosteroids	Placebo/no treatment/standard care	Mortality
CD004417	Delayed antibiotics for respiratory infections	Issue 3; 2007	10.1002/14651858.CD004417.pub3	15.01	Delayed antibiotics	Immediate antibiotics	Patient satisfaction: Delayed versus Immediate Antibiotics
CD004434	Endothelin receptor antagonists for pulmonary arterial hypertension	Issue 3; 2006	10.1002/14651858.CD004434.pub3	1.02	ERAs	Placebo	proportion of patients with improved functional class
CD004437	Thrombolytic therapy for pulmonary embolism	Issue 2; 2006	10.1002/14651858.CD004437.pub2	1.01	Thrombolytic (rt-PA, streptokinase, urokinase)	Heparin	All cause mortality
CD004472	Preoperative intra-aortic balloon pumps in patients undergoing coronary artery bypass grafting	Issue 1; 2007	10.1002/14651858.CD004472.pub2	1.01	Preoperative IABP	No preop IABP (no treatment)	In-hospital death
CD004509	Testosterone for peri and postmenopausal women	Issue 4; 2005	10.1002/14651858.CD004509.pub2	14.11	HRT + testosterone	HRT alone	Discontinuation rate (sbgrp 1: all studies)
CD004549	Wound drainage for caesarean section	Issue 1; 2005	10.1002/14651858.CD004549.pub2	1.02	Drain	No drain	Wound complications
CD004661	Magnesium sulphate for women at risk of preterm birth for neuroprotection of the fetus	Issue 1; 2009	10.1002/14651858.CD004661.pub3	1.01	Magnesium	No magnesium	Paediatric mortality (fetal and later)
CD004678	Glatiramer acetate for multiple sclerosis	Issue 1; 2004	10.1002/14651858.CD004678	3.03	Glatiramer acetate	Placebo	Adverse effects causing treatment withdrawal
CD004720	Screening for prostate cancer	Issue 3; 2006	10.1002/14651858.CD004720.pub2	1.01	Screening	No screening	Prostate cancer-specific mortality (sub-group analysis risk of bias)
CD004736	Effects and safety of preventive oral iron or iron+folic acid supplementation for women during pregnancy	Issue 3; 2006	10.1002/14651858.CD004736.pub2	1.09	Daily iron alone	no iron/placebo	Anaemia at term (Hb less than 110 g/L) (ALL)
CD004816	Statins for the primary prevention of cardiovascular disease	Issue 1; 2011	10.1002/14651858.CD004816.pub4	2.04	Statin Therapy Group	Usual Care or Placebo	Total Number of CHD Events
CD004863	Early erythropoietin for preventing red blood cell transfusion in preterm and/or low birth weight infants	Issue 3; 2006	10.1002/14651858.CD004863.pub2	1.01	EPO	placebo or no treatment	Use of one or more red blood cell transfusions (low

							and high dose of EPO)
CD004868	Late erythropoietin for preventing red blood cell transfusion in preterm and/or low birth weight infants	Issue 3; 2006	10.1002/14651858.CD004868.pub2	1.01	Late EPO	placebo or no intervention	Use of one or more red blood cell transfusions (low and high dose of EPO)
CD004878	Glucocorticoids for acute viral bronchiolitis in infants and young children	Issue 10; 2010	10.1002/14651858.CD004878.pub3	1.01	Glucocorticoid	Placebo	Admissions (days 1 and 7) (outpatients) - review primary outcome
CD004888	Antiviral treatment for chronic hepatitis C in patients with human immunodeficiency virus	Issue 1; 2010	10.1002/14651858.CD004888.pub2	1.01	PEGinterferon + ribavirin	Interferon + ribavirin	Sustained virological response
CD004907	Package of care for active management in labour for reducing caesarean section rates in low-risk women	Issue 4; 2008	10.1002/14651858.CD004907.pub2	1.01	Active management	Routine care	Caesarean section rate - all women
CD004958	Single or double-level anterior interbody fusion techniques for cervical degenerative disc disease	Issue 1; 2011	10.1002/14651858.CD004958.pub2	1.08	Fusion + discectomy	discectomy alone	No Fusion
CD005011	Recombinant factor VIIa for the prevention and treatment of bleeding in patients without haemophilia	Issue 2; 2007	10.1002/14651858.CD005011.pub2	3.01	rFVIIa	Placebo	Total thromboembolic events
CD005044	Quinine for muscle cramps	Issue 4; 2004	10.1002/14651858.CD005044	1.09	Quinine	Placebo	Participants suffering major adverse events
CD005052	Shengmai (a traditional Chinese herbal medicine) for heart failure	Issue 4; 2007	10.1002/14651858.CD005052.pub2	1.01	Shengmai+usual	usual	Lack of improvement in heart failure (NYHA class improved < I class or worsening of heart failure)
CD005128	Interventions for preventing and treating kidney disease in Henoch-Schonlein Purpura (HSP)	Issue 3; 2009	10.1002/14651858.CD005128.pub2	1.01	Prednisone	Placebo/supportive treatment	Persistent kidney disease at any time after treatment
CD005188	Interventions to increase influenza vaccination rates of those 60 years and older in the community	Issue 2; 2005	10.1002/14651858.CD005188	1.02	Reminder & recall: phone call or letter	No intervention	Increasing community demand
CD005215	Interventions for preventing unintended pregnancies among adolescents	Issue 4; 2009	10.1002/14651858.CD005215.pub2	1.06	Multiple Intervention	No intervention / routine care	Use of birth control methods-Individually RCT
CD005291	Preimplantation genetic screening for abnormal number of chromosomes (aneuploidies) in in vitro fertilisation or intracytoplasmic sperm injection	Issue 1; 2006	10.1002/14651858.CD005291.pub2	1.01	Preimplantation genetic screening	No screening	Live birth rate per woman randomised

CD005307	Addition of long-acting beta2-agonists to inhaled steroids as first line therapy for persistent asthma in steroid-naive adults and children	Issue 4; 2009	10.1002/14651858.CD005307.pub2	1.01	ICS + LABA	ICS alone	# patients with exacerbations requiring systemic steroids
CD005341	Granulocyte transfusions for preventing infections in patients with neutropenia or neutrophil dysfunction	Issue 3; 2005	10.1002/14651858.CD005341	1.01	Granulocytes	No granulocytes	Overall mortality
CD005354	Recombinant versus urinary gonadotrophin for ovarian stimulation in assisted reproductive technology cycles	Issue 2; 2011	10.1002/14651858.CD005354.pub2	1.08	rFSH	Urinary gonadotrophins	Clinical pregnancy by urinary gonadotrophin
CD005415	Proton pump inhibitor treatment initiated prior to endoscopic diagnosis in upper gastrointestinal bleeding	Issue 4; 2006	10.1002/14651858.CD005415.pub2	1.01	Proton pump inhibitor	placebo, an H2RA or no treatment	Mortality - 30 days or at point closest to 30 days
CD005431	Medical interventions for traumatic hyphema	Issue 3; 2005	10.1002/14651858.CD005431	1.05	Antifibrinolytics	Placebo	Risk of secondary hemorrhage
CD005442	Bile acids for liver-transplanted patients	Issue 3; 2005	10.1002/14651858.CD005442	1.01	bile acids	placebo/ no intervention	All-cause mortality at maximum follow-up
CD005496	Probiotics for prevention of necrotizing enterocolitis in preterm infants	Issue 1; 2008	10.1002/14651858.CD005496.pub2	1.01	Probiotics	Placebo or no treatment	Severe necrotising enterocolitis (stage II-III)
CD005535	Addition of long-acting beta2-agonists to inhaled corticosteroids versus same dose inhaled corticosteroids for chronic asthma in adults and children	Issue 4; 2005	10.1002/14651858.CD005535	1.01	LABA + ICS	ICS alone + placebo	# patients with exacerbations requiring oral steroids
CD005571	Systemic antimicrobial prophylaxis for percutaneous endoscopic gastrostomy	Issue 4; 2006	10.1002/14651858.CD005571.pub2	3.01	antibiotic	placebo/none/antiseptic	Peristomal infection
CD005967	Artesunate versus quinine for treating severe malaria	Issue 4; 2007	10.1002/14651858.CD005967.pub2	1.01	Artesunate	Quinine	Death: participant age
CD005981	Direct thrombin inhibitors versus vitamin K antagonists or low molecular weight heparins for prevention of venous thromboembolism following total hip or knee replacement	Issue 4; 2010	10.1002/14651858.CD005981.pub2	2.04	Direct Thrombin Inhibitors (any dose)	LMWH	All-cause Mortality events combined doses in THR+TKR
CD006009	Dopamine agonists for restless legs syndrome	Issue 2; 2006	10.1002/14651858.CD006009	1.07	Dopamine agonists	Placebo	Number of dropouts due to adverse events
CD006015	Finasteride for benign prostatic hyperplasia	Issue 10; 2010	10.1002/14651858.CD006015.pub3	1.19	Finasteride 5 mg	Placebo	Study discontinuations (f/u = 1 yr)
CD006103	Nicotine receptor partial agonists for smoking cessation	Issue 12; 2010	10.1002/14651858.CD006103.pub4	1.01	Varenicline (1.0mg 2/d)	Placebo	Continuous abstinence at longest follow up (24+ weeks)
CD006105	Metformin treatment before and during IVF or ICSI in women with polycystic ovary syndrome	Issue 2; 2009	10.1002/14651858.CD006105.pub2	1.04	Metformin	Placebo	Incidence of OHSS

CD006114	Fluvoxamine versus other anti-depressive agents for depression	Issue 3; 2006	10.1002/14651858.CD006114	1.01	Fluvoxamine	TCAs	Response (acute phase): Primary outcome
CD006117	Sertraline versus other antidepressive agents for depression	Issue 1; 2010	10.1002/14651858.CD006117.pub3	1.01	Sertraline	TCAs (Subgrp: amitriptyline)	Failure to respond at endpoint (6 - 12 weeks): Sertraline versus TCAs
CD006117	Sertraline versus other antidepressive agents for depression	Issue 1; 2010	10.1002/14651858.CD006117.pub3	4.01	Sertraline	TCAs (Subgrp: imipramine)	Failure to remission at endpoint (6 - 12 weeks): Sertraline versus TCAs
CD006117	Sertraline versus other antidepressive agents for depression	Issue 1; 2010	10.1002/14651858.CD006117.pub3	13.01	Sertraline	TCAs (Subgrp: clomipramine)	SE Participants with at least one TEAE (Treatment Emergent Adverse Event?)
CD006353	Use of plastic adhesive drapes during surgery for preventing surgical site infection	Issue 4; 2007	10.1002/14651858.CD006353.pub2	1.01	Adhesive drape	No adhesive drape	Surgical site infection (all wound classifications)
CD006369	Oral paliperidone for schizophrenia	Issue 2; 2008	10.1002/14651858.CD006369.pub2	1.01	Paliperidone	Placebo	Leaving the study early
CD006466	Oral anticoagulation in patients with cancer who have no therapeutic or prophylactic indication for anticoagulation	Issue 2; 2007	10.1002/14651858.CD006466	1.02	Warfarin	No warfarin	Death at 1 year
CD006468	Anticoagulation for patients with cancer and central venous catheters	Issue 2; 2011	10.1002/14651858.CD006468.pub3	1.01	Heparin	No heparin (no treatment)	Death
CD006480	Mono and multifaceted inhalant and/or food allergen reduction interventions for preventing asthma in children at high risk of developing asthma	Issue 3; 2009	10.1002/14651858.CD006480.pub2	1.02	Allergen reduction interventions (Mono-faceted intervention)	Placebo or usual care	Current diagnosis asthma (ITT)
CD006529	Milnacipran versus other antidepressive agents for depression	Issue 2; 2007	10.1002/14651858.CD006529	10.01	Milnacipran	TCAs	Dropouts due to inefficiency
CD006536	Stem cell treatment for acute myocardial infarction	Issue 4; 2008	10.1002/14651858.CD006536.pub2	1.01	BMSC (Stem cells)	no BMSC	Mortality
CD006537	Laser treatment of drusen to prevent progression to advanced age-related macular degeneration	Issue 2; 2007	10.1002/14651858.CD006537	1.01	Photocoagulation	No treatment	Development of CNV
CD006626	Risperidone versus other atypical antipsychotics for schizophrenia	Issue 3; 2007	10.1002/14651858.CD006626	3.01	RISPERIDONE	CLOZAPINE	Global state: 1a. No clinically significant response (as defined by the original studies)
CD006632	Clinical pathways: effects on professional practice, patient outcomes, length of stay and hospital costs	Issue 3; 2010	10.1002/14651858.CD006632.pub2	2.2	Clinical pathway	Usual care	Hospital readmission up to 6 months

CD006649	Anticoagulation for the initial treatment of venous thromboembolism in patients with cancer	Issue 2; 2011	10.1002/14651858.CD006649.pub3	1.01	LMWH	UFH	Death at 3 months
CD006652	Parenteral anticoagulation in patients with cancer who have no therapeutic or prophylactic indication for anticoagulation	Issue 1; 2011	10.1002/14651858.CD006652.pub2	1.01	Heparin	Placebo	Mortality at 12 months
CD006654	Olanzapine versus other atypical antipsychotics for schizophrenia	Issue 3; 2010	10.1002/14651858.CD006654.pub2	3.01	OLANZAPINE	CLOZAPINE	Global state: 1a. no clinically significant response (as defined by the original studies)
CD006690	Chinese herbal medicines for people with impaired glucose tolerance or impaired fasting blood glucose	Issue 3; 2007	10.1002/14651858.CD006690	1.02	Herbs + lifestyle	Lifestyle alone	Incidence of diabetes (n)
CD006706	Interventions for the prevention and treatment of herpes simplex virus in patients being treated for cancer	Issue 3; 2007	10.1002/14651858.CD006706	1.03	Aciclovir	Placebo	Viral isolates (by mode of administration)
CD006739	Dipeptidyl peptidase-4 (DPP-4) inhibitors for type 2 diabetes mellitus	Issue 2; 2008	10.1002/14651858.CD006739.pub2	1.02	Sitagliptin	Placebo/another single hypoglycemic drug/no treatment	Adverse events [n]
CD006739	Dipeptidyl peptidase-4 (DPP-4) inhibitors for type 2 diabetes mellitus	Issue 2; 2008	10.1002/14651858.CD006739.pub2	2.02	Vildagliptin	Control	Adverse events [n]
CD006743	Effect of early treatment with anti-hypertensive drugs on short and long-term mortality in patients with an acute cardiovascular event	Issue 4; 2009	10.1002/14651858.CD006743.pub2	1.02	Nitrates	Placebo/no treatment	All-cause mortality at 10 days
CD006743	Effect of early treatment with anti-hypertensive drugs on short and long-term mortality in patients with an acute cardiovascular event	Issue 4; 2009	10.1002/14651858.CD006743.pub2	3.02	Beta-blockers	Placebo/no treatment	All-cause mortality at 10 days
CD006743	Effect of early treatment with anti-hypertensive drugs on short and long-term mortality in patients with an acute cardiovascular event	Issue 4; 2009	10.1002/14651858.CD006743.pub2	4.02	Calcium-channel blockers	Placebo/no treatment	All-cause mortality at 10 days
CD006772	Service organisation for the secondary prevention of ischaemic heart disease in primary care	Issue 4; 2007	10.1002/14651858.CD006772	1.15	Control (normal care)	Intervention (Service organisation)	Prescribed anti-platelet medication at end of study
CD006794	Early amniotomy and early oxytocin for prevention of, or therapy for, delay in first stage spontaneous labour compared with routine care	Issue 4; 2007	10.1002/14651858.CD006794	1.01	Early amniotomy and early oxytocin	routine care	Caesarean section rate
CD006804	Miniport versus standard ports for laparoscopic cholecystectomy	Issue 3; 2010	10.1002/14651858.CD006804.pub2	1.06	Miniport-laparoscopic cholecystectomy	Standard port laparoscopic cholecystectomy	Conversion to open cholecystectomy
CD006829	Combined corticosteroid and long-acting beta-agonist in one inhaler versus long-acting beta-agonists for chronic obstructive pulmonary disease	Issue 4; 2007	10.1002/14651858.CD006829	1.03	Combination inhalers (steroid + LABA)	Long-acting beta-agonists alone	Pneumonia
CD006849	Platinum versus non-platinum chemotherapy regimens for small cell lung cancer	Issue 4; 2008	10.1002/14651858.CD006849.pub2	1.01	Platinum regime	Non-platinum regime	6-month survival

CD006913	Psychological interventions for treatment of inflammatory bowel disease	Issue 1; 2008	10.1002/14651858.CD006913	1.11	Psychotherapy	no treatment/ other type of therapy, including other psychosocial / sham / standard medical therapy	Not in remission at/during month 9 to 18
CD006918	Risperidone versus placebo for schizophrenia	Issue 1; 2008	10.1002/14651858.CD006918	1.06	Risperidone	Placebo	Mental state: 1c. No specific degree of response (<20% decrease PANSS/BPRS total change)
CD006920	Acupuncture and assisted conception	Issue 4; 2008	10.1002/14651858.CD006920.pub2	3.03	Acupuncture	No accupuncture	Clinical Pregnancy Rate
CD006920	Acupuncture and assisted conception	Issue 4; 2008	10.1002/14651858.CD006920.pub2	4.03	Acupuncture	Sham or no treatment	Clinical pregnancy
CD006922	Regular treatment with salmeterol and inhaled steroids for chronic asthma: serious adverse events	Issue 1; 2008	10.1002/14651858.CD006922	1.01	Salmeterol and ICS	ICS	All-cause mortality
CD006923	Regular treatment with formoterol for chronic asthma: serious adverse events	Issue 4; 2008	10.1002/14651858.CD006923.pub2	2.01	Formoterol	Placebo	Non-fatal serious adverse events (adults & children)
CD007115	Duloxetine for treating painful neuropathy or chronic pain	Issue 4; 2009	10.1002/14651858.CD007115.pub2	3.06	Duloxetine	Placebo	Adverse event leading to cessation
CD007125	Multidisciplinary rehabilitation for older people with hip fractures	Issue 2; 2008	10.1002/14651858.CD007125	1.01	Multidisciplinary inpatient rehabilitation	Usual care	'Poor outcome' (long-term follow-up)
CD007187	Blood pressure lowering efficacy of diuretics as second-line therapy for primary hypertension	Issue 2; 2008	10.1002/14651858.CD007187	5.04	Combination	Monotherapy	Withdrawals due to adverse events
CD007223	Medical treatments for incomplete miscarriage (less than 24 weeks)	Issue 3; 2008	10.1002/14651858.CD007223	2.02	Misoprostol	Surgery	Surgical evacuation
CD007228	Structured telephone support or telemonitoring programmes for patients with chronic heart failure	Issue 8; 2010	10.1002/14651858.CD007228.pub2	1.01	structured telephone support	Usual Care	All-cause mortality (full peer-reviewed publications only): structured telephone support vs usual care
CD007253	Interventions for smoking cessation and reduction in individuals with schizophrenia	Issue 6; 2010	10.1002/14651858.CD007253.pub2	1.01	Bupropion	Placebo	Abstinence at 6-month follow-up (primary outcome)
CD007275	Effects of communicating DNA-based disease risk estimates on risk-reducing behaviours	Issue 3; 2008	10.1002/14651858.CD007275	1.01	Communicating DNA-based disease risk estimates to motivate behavour change	No genetic tests risk estimates	Smoking
CD007277	Abatacept for rheumatoid arthritis	Issue 4; 2009	10.1002/14651858.CD007277.pub2	1.25	Abatacept	Placebo	All withdrawals

CD007339	Pentoxifylline for alcoholic hepatitis	Issue 3; 2008	10.1002/14651858.CD007339	1.01	Pentoxifylline	Placebo/no treatment	Mortality using the fixed effect model
CD007345	Antibiotic prophylaxis for patients undergoing elective endoscopic retrograde cholangiopancreatography	Issue 10; 2010	10.1002/14651858.CD007345.pub2	1.02	Antibiotic prophylaxis	Placebo	Acute cholangitis
CD007411	Antioxidants for male subfertility	Issue 1; 2011	10.1002/14651858.CD007411.pub2	1.02	Antioxidants	No treatment/placebo	Pregnancy rate per couple randomised
CD007482	Antibiotic prophylaxis versus no prophylaxis for preventing infection after cesarean section	Issue 4; 2008	10.1002/14651858.CD007482	1.02	Antibiotic	No antibiotic	Maternal wound infection
CD007503	Antidepressants for depression in physically ill people	Issue 3; 2010	10.1002/14651858.CD007503.pub2	1.02	Antidepressants	Placebo	Response to treatment (6-8 weeks). Antidepressants versus placebo
CD007613	Erythropoiesis-stimulating agents for anaemia in chronic heart failure patients	Issue 1; 2010	10.1002/14651858.CD007613.pub2	1.16	Erythropoiesis-stimulating agents (ESA)	Control	All-cause mortality
CD007683	Laparoscopy for the management of acute lower abdominal pain in women of childbearing age	Issue 2; 2009	10.1002/14651858.CD007683	1.02	Laparoscopic diagnosis	Open surgery diagnosis	Any adverse events
CD007710	Pain relief for outpatient hysteroscopy	Issue 2; 2009	10.1002/14651858.CD007710	1.04	Analgesic (sbgrp 1: Local Anaesthetics)	Placebo or no comparison	Vasovagal reaction (fainting)
CD007749	Antioxidant supplements for liver diseases	Issue 2; 2009	10.1002/14651858.CD007749	1.01	Antioxidants	placebo or no intervention	All-cause mortality
CD007780	Cisapride for Intestinal Constipation	Issue 2; 2009	10.1002/14651858.CD007780	3.01	Cisapride	Placebo	Side effects
CD007781	Laparoscopic versus open surgical techniques for ventral or incisional hernia repair	Issue 2; 2009	10.1002/14651858.CD007781	1.01	Laparoscopic repair	Conventional/open repair	Hernia recurrence
CD007798	Clinically-indicated replacement versus routine replacement of peripheral venous catheters	Issue 2; 2009	10.1002/14651858.CD007798	1.02	Clinically indicated replacement	Routine replacement	Phlebitis all studies
CD007906	Intensive case management for severe mental illness	Issue 10; 2010	10.1002/14651858.CD007906.pub2	1.02	INTENSIVE CASE MANAGEMENT	STANDARD CARE	Service use: 2. Not remaining in contact with psychiatric services
CD007949	Addition of long-acting beta-agonists to inhaled corticosteroids for chronic asthma in children	Issue 3; 2009	10.1002/14651858.CD007949	1.04	LABA + ICS	ICS alone	Total # withdrawals
CD007950	Effects and safety of periconceptional folate supplementation for preventing birth defects	Issue 3; 2009	10.1002/14651858.CD007950	1.11	Folic acid	No treatment/other micronutrients/placebo	Miscarriage (ALL)
CD008046	Gonadotropin-releasing hormone agonist versus HCG for oocyte triggering in antagonist assisted reproductive technology cycles	Issue 11; 2010	10.1002/14651858.CD008046.pub2	1.02	GnRH agonist	HCG (standard medication for this purpose)	OHSS incidence per women randomised

CD008121	Second-generation antipsychotics for major depressive disorder and dysthymia	Issue 4; 2009	10.1002/14651858.CD008121	5.01	Olanzapine (+ antidepessants)	Placebo (+antidepressants)	No clinically important response - as defined by original study
CD008141	Second-generation antipsychotics for obsessive compulsive disorder	Issue 4; 2009	10.1002/14651858.CD008141	2.01	Quetiapine (+antidepressants)	Placebo (+antidepressants)	No clinically important response to treatment (as defined by the original study)
CD008331	Tocilizumab for rheumatoid arthritis	Issue 1; 2010	10.1002/14651858.CD008331	14.02	Placebo	Tocilizumab	ACR 50% improvement
CD008370	Somatostatin analogues for pancreatic surgery	Issue 2; 2010	10.1002/14651858.CD008370	1.01	Somatostatin analogues	No Somatostatin analogues	Perioperative mortality
CD008407	Paracetamol/acetaminophen (single administration) for perineal pain in the early postpartum period	Issue 3; 2010	10.1002/14651858.CD008407	1.01	Paracetamol	Placebo	Adequate pain relief as reported by women
CD008414	Oral 5-aminosalicylic acid for maintenance of surgically-induced remission in Crohn's disease	Issue 3; 2010	10.1002/14651858.CD008414	1.01	5-ASA	Placebo	Relapse, drop-outs classed as relapse, fixed effects model
CD008418	Formoterol versus short-acting beta-agonists as relief medication for adults and children with asthma	Issue 3; 2010	10.1002/14651858.CD008418	1.04	Formoterol (long-acting BA)	Short-acting beta-agonist (standard relief medication)	Patients with a serious adverse event (all-cause)
CD008495	Methotrexate monotherapy versus methotrexate combination therapy with non-biologic disease modifying anti-rheumatic drugs for rheumatoid arthritis	Issue 4; 2010	10.1002/14651858.CD008495	1.09	Methotrexat + other non-bilogic drugs	Methotrexate mono	Combined withdrawal due to lack of efficacy or toxicity (stratified by regimen)
CD008521	Vaccines for preventing rotavirus diarrhoea: vaccines in use	Issue 5; 2010	10.1002/14651858.CD008521	1.13	Rotarix	Placebo	Serious adverse events
CD008521	Vaccines for preventing rotavirus diarrhoea: vaccines in use	Issue 5; 2010	10.1002/14651858.CD008521	2.09	RotaTeq	Placebo	Reactogenicity: fever
CD008567	General physical health advice for people with serious mental illness	Issue 7; 2010	10.1002/14651858.CD008567	1.05	PHYSICAL HEALTH ADVICE	STANDARD CARE	Leaving the study early
CD008603	Oral vaccines for preventing cholera	Issue 7; 2010	10.1002/14651858.CD008603	6.03	Vaccine (Whole cell plus recombinant B subunit (WC-rBS))	Placebo	Adverse events (Subgroup 6.3.3: Diarrhoea)
CD008603	Oral vaccines for preventing cholera	Issue 7; 2010	10.1002/14651858.CD008603	9.01	Vaccine (Live attenuated vaccines: CVD 103-HgR)	Placebo	Adverse events (Subgroup 9.1.1: Diarrhoea)
CD008960	Phyllanthus species for chronic hepatitis B virus infection	Issue 1; 2011	10.1002/14651858.CD008960	1.04	Phyllantus	Control	Number of patients with detectable serum HBeAg (end of treatment)

* Meta-analysis number within the review (the first number refers to the comparison number and the second is the meta-analysis number within the comparison)

Web Figure 2. Number of Trials With Each Combination of the Four Risk of Bias Domain Judgements by Type of Outcome Measure

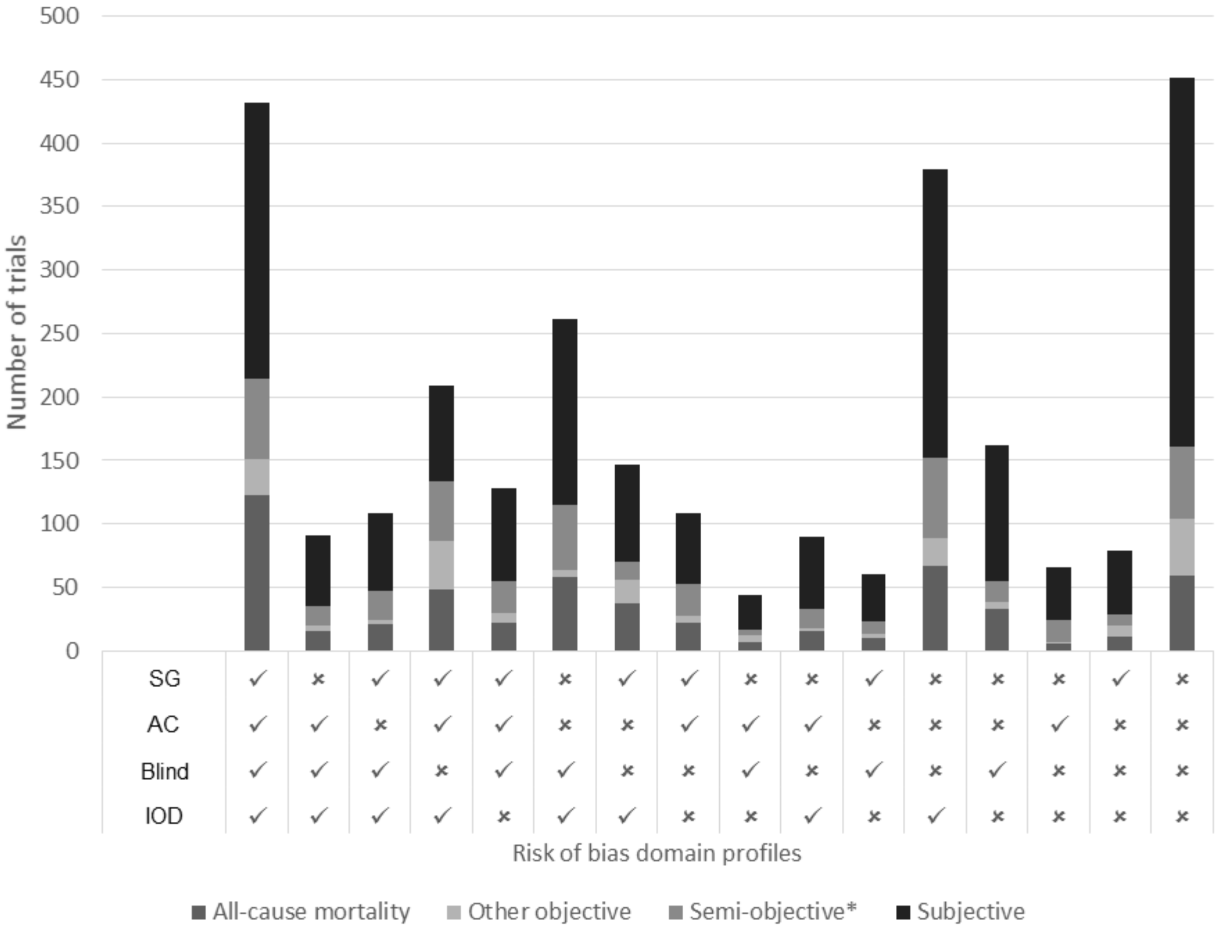

✓= Low risk of bias; × = High or unclear risk of bias; SG = Sequence generation; AC = Allocation concealment; Blind = Blinding; IOD = Incomplete outcome data. * Semi-objective outcomes are those that are thought to be accurately assessed but potentially influenced by clinician/patient judgment (e.g. hospital admissions, duration of hospitalization, withdrawals, caesarian section etc.)

Web Figure 3. Estimated Ratios of Odds Ratios and Effects on Heterogeneity Associated With Risk of Bias Judgements for Each Domain Independently, According to Type of Outcome Measure: Univariable Analyses (Model A)

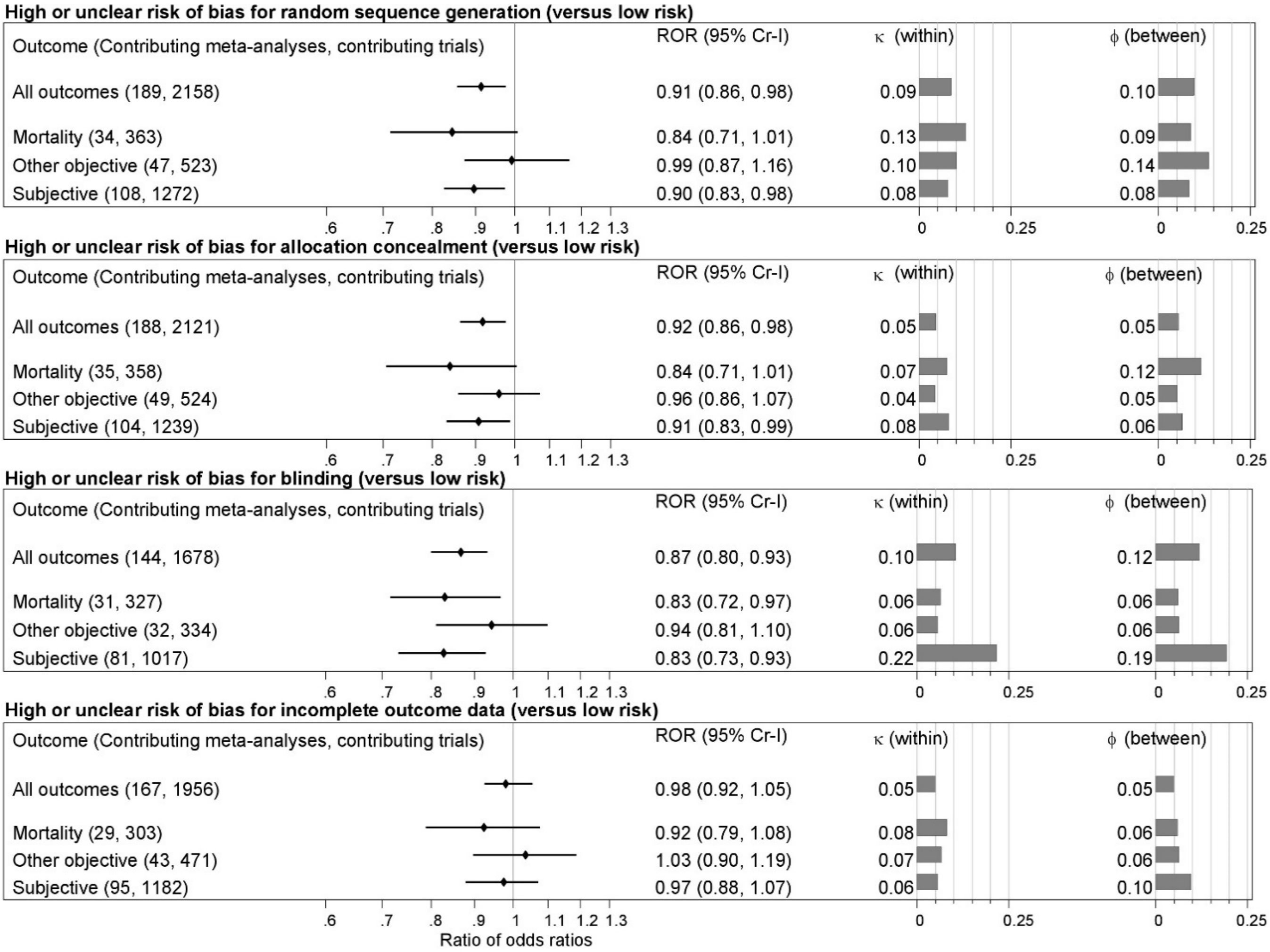

This figure corresponds to Table 4 in the main paper. Objective outcomes and semi-objective outcomes combined. ROR = ratio of risk ratios; CrI = credible interval; κ – measure of within meta-analysis heterogeneity; φ – measure of between meta-analysis heterogeneity.

Web Figure 4. Estimated Ratios of Odds Ratios and Effects on Heterogeneity From Multivariable Analyses of Associations With Risk of Bias Judgements for Each Domain, Adjusted for the Effect of the Other Three Domains (Model B)

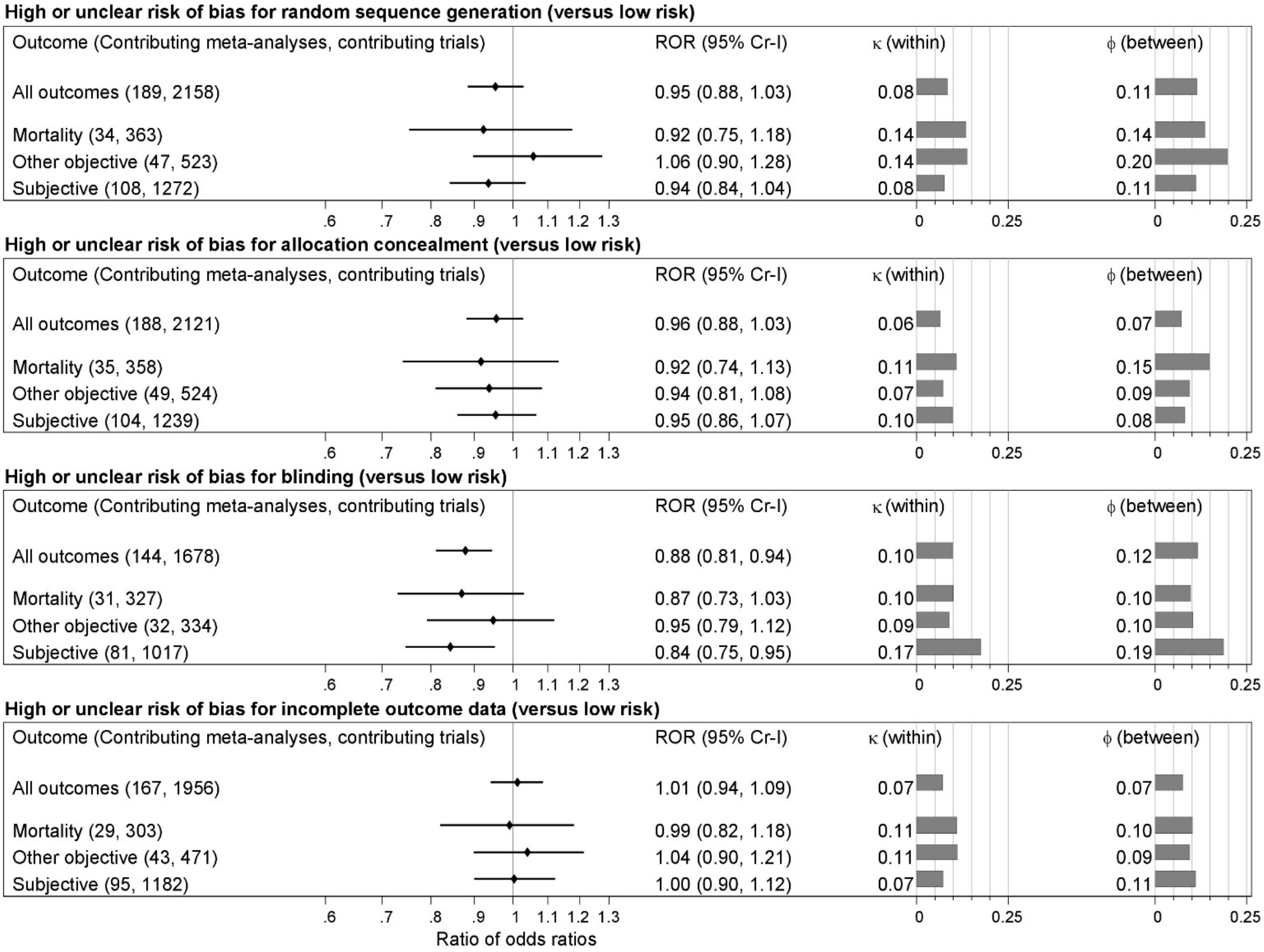

This figure corresponds to Table 5 in the main paper. Objective outcomes and semi-objective outcomes combined. Analyses for each bias domain were adjusted for risk of bas judgements for the other three domains. ROR = ratio of risk ratios; CrI = credible interval; κ – measure of within meta-analysis heterogeneity; φ – measure of between meta-analysis heterogeneity.

Web Table 1. Estimated Ratios of Odds Ratios and Between-Meta-Analysis Heterogeneity in Mean Bias Associated With Risk of Bias Judgements, According to Type of Outcome Measure: Univariable Sensitivity Analyses for High Risk of Bias Compared to Low or Unclear Risk of Bias

Risk-of-Bias Domain and Outcome	Contributing Meta-Analyses (Trials)	Average Bias		Meta-Analyses Contributing to κ Estimation	Within-Meta-Analysis Heterogeneity		Between-Meta-Analysis Heterogeneity	
		ROR	95% CrI		κ	95% CrI	φ	95% CrI
All outcomes: High risk of bias vs. low/unclear risk of bias								
Sequence generation	39 (511)	0.86	0.67, 1.09	12	0.35	0.02, 0.77	0.32	0.03, 0.67
Allocation concealment	60 (790)	0.92	0.78, 1.06	28	0.06	0.01, 0.27	0.22	0.03, 0.43
Blinding	117 (1358)	0.87	0.79, 0.95	67	0.18	0.02, 0.35	0.11	0.01, 0.28
Incomplete outcome data	116 (1465)	1.06	0.97, 1.15	65	0.06	0.01, 0.21	0.08	0.01, 0.23

ROR = ratio of odds ratios; CrI = credible interval.

Web Table 2. Estimated Ratios of Odds Ratios and Between-Meta-Analysis Heterogeneity in Mean Bias Associated With Risk of Bias Judgements, According to Type of Outcome Measure: Univariable Sensitivity Analyses for Meta-Analyses With Other Objective and Semi-Objective Outcomes and for High Risk of Bias Compared to Low Or Unclear Risk of Bias

Risk-of-Bias Domain and Outcome	Contributing Meta-Analyses (Trials)	Average Bias		Meta-Analyses Contributing to κ Estimation	Within-Meta-Analysis Heterogeneity		Between-Meta-Analysis Heterogeneity	
		ROR	95% CrI		κ	95% CrI	φ	95% CrI
Sequence generation: High/unclear risk of bias vs. low risk of bias								
Other objective	18 (187)	0.85	0.67, 1.09	13	0.08	0.01, 0.40	0.09	0.01, 0.50
Semi-objective	29 (336)	1.08	0.91, 1.34	25	0.12	0.01, 0.36	0.18	0.02, 0.45
Allocation concealment: High/unclear risk of bias vs. low risk of bias								
Other objective	17 (167)	0.95	0.78, 1.15	14	0.06	0.01, 0.25	0.07	0.01, 0.36
Semi-objective	32 (357)	0.96	0.83, 1.12	26	0.05	0.01, 0.17	0.07	0.01, 0.28
Blinding: High/unclear risk of bias vs. low risk of bias								
Other objective	13 (105)	0.97	0.71, 1.31	12	0.08	0.01, 0.39	0.11	0.01, 0.61
Semi-objective	19 (229)	0.93	0.76, 1.12	12	0.06	0.01, 0.24	0.07	0.01, 0.36
Incomplete outcome data: High/unclear risk of bias vs. low risk of bias								
Other objective	16 (167)	0.94	0.72, 1.22	10	0.08	0.01, 0.40	0.08	0.01, 0.47
Semi-objective	27 (304)	1.11	0.92, 1.30	18	0.08	0.01, 0.31	0.06	0.01, 0.29

ROR = ratio of odds ratios; CrI = credible interval.

Web Table 3. Estimated Ratios of Odds Ratios and Between-Meta-Analysis Heterogeneity in Mean Bias Associated With Risk of Bias Judgments, According to Type of Outcome Measure: Multivariable Analyses With Interactions

Risk of Bias Domain and Outcome	Average Bias		Meta-Analyses Contributing to κ Estimation	Within-Meta-Analysis Heterogeneity		Between-Meta-Analysis Heterogeneity	
	ROR	95% CrI		κ	95% CrI	φ	95% CrI
Sequence Generation							
All	0.98	0.84, 1.11	142	0.06	0.01, 0.16	0.09	0.02, 0.22
Mortality	1.08	0.75, 1.62	27	0.11	0.03, 0.29	0.12	0.02, 0.35
Other objective/semi-objective	1.13	0.84, 1.64	38	0.11	0.02, 0.29	0.17	0.03, 0.41
Subjective/Mixed	0.94	0.79, 1.09	77	0.07	0.02, 0.16	0.09	0.02, 0.22
Allocation Concealment							
All	0.91	0.81, 1.03	139	0.06	0.02, 0.14	0.08	0.02, 0.16
Mortality	0.85	0.59, 1.17	27	0.10	0.02, 0.28	0.16	0.03, 0.45
Other objective/semi-objective	0.88	0.71, 1.16	40	0.07	0.02, 0.18	0.10	0.02, 0.26
Subjective/Mixed	0.92	0.80 1.09	72	0.08	0.02, 0.22	0.08	0.01, 0.20
Blinding							
All	0.92	0.84, 1.03	105	0.08	0.02, 0.19	0.07	0.02, 0.18
Mortality	0.90	0.73, 1.13	25	0.09	0.02, 0.25	0.10	0.02, 0.30
Other objective/semi-objective	0.99	0.82, 1.25	24	0.08	0.01, 0.21	0.10	0.02, 0.33
Subjective/Mixed	0.89	0.74, 1.03	56	0.11	0.03, 0.27	0.13	0.03, 0.29
Interaction Between Allocation Concealment and Sequence Generation							
All	0.93	0.77, 1.16	109	0.09	0.02, 0.20	0.09	0.02, 0.20
Mortality	0.81	0.43, 1.38	20	0.16	0.03, 0.49	0.15	0.03, 0.45
Other objective/semi-objective	0.94	0.66, 1.57	32	0.09	0.02, 0.26	0.12	0.02, 0.35
Subjective/Mixed	0.89	0.70, 1.10	57	0.10	0.01, 0.25	0.09	0.02, 0.19
Interaction Between Allocation Concealment and Blinding							
All	0.84	0.74, 0.96	80	0.13	0.02, 0.29	0.09	0.02, 0.20
Mortality	0.84	0.62, 1.19	19	0.15	0.03, 0.45	0.15	0.03, 0.51
Other objective/semi-objective	0.92	0.71, 1.19	19	0.12	0.03, 0.33	0.11	0.02, 0.34
Subjective/Mixed	0.82	0.69, 1.01	42	0.14	0.03, 0.34	0.09	0.02, 0.23
Interaction Between Blinding and Sequence Generation							
All	0.77	0.66, 0.91	81	0.10	0.02, 0.25	0.11	0.03, 0.23
Mortality	0.76	0.48, 1.20	19	0.15	0.02, 0.49	0.12	0.02, 0.36
Other objective/semi-objective	0.93	0.68, 1.31	20	0.13	0.03, 0.41	0.14	0.03, 0.37
Subjective/Mixed	0.72	0.58, 0.93	42	0.10	0.02, 0.27	0.12	0.02, 0.30
Incomplete Outcome Data							
All	1.00	0.94, 1.08	112	0.07	0.01, 0.15	0.07	0.01, 0.17
Mortality	1.00	0.81, 1.20	19	0.11	0.03, 0.30	0.11	0.02, 0.30
Other objective/semi-objective	1.03	0.88, 1.20	28	0.12	0.03, 0.29	0.10	0.02, 0.30
Subjective/Mixed	1.01	0.90, 1.12	65	0.07	0.02, 0.16	0.10	0.03, 0.22

Number of contributing meta-analyses (trials): all outcomes 222 (2403); mortality 42 (429); Other objective/semi-objective 54 (568); Subjective/mixed 126 (1406). ROR = ratio of odds ratios; CrI = credible interval.
